# Unraveling the Versatility of Carbon Black – Polylactic Acid (CB/PLA) 3D‐Printed Electrodes via Sustainable Electrochemical Activation

**DOI:** 10.1002/smtd.202402214

**Published:** 2025-03-13

**Authors:** Anastasios V. Papavasileiou, Lukáš Děkanovský, Levna Chacko, Bing Wu, Jan Luxa, Jakub Regner, Jan Paštika, Dana Koňáková, Zdeněk Sofer

**Affiliations:** ^1^ Department of Inorganic Chemistry University of Chemistry and Technology Prague Technicka 5 Prague 6 16628 Czech Republic; ^2^ Department of Materials Engineering and Chemistry Faculty of Civil Engineering Czech Technical University Thakurova 7 Prague 6 160 00 Czech Republic

**Keywords:** 3D printed electrodes, aqueous battery, electrochemical activation, grid‐layout electrode, sensing

## Abstract

Commercially available conductive filaments are not designed for electrochemical applications, resulting in 3D printed electrodes with poor electrochemical behavior, restricting their implementation in energy and sensing technologies. The proper selection of an activation method can unlock their use in advanced applications. In this work, rectangular electrodes made from carbon black – polylactic acid (CB/PLA) filament are 3D printed with different layouts (grid and compact) and then activated using a highly reproducible eco‐compatible electrochemical (EC) treatment. The electrodes are characterized for their morphological, structural, and electrochemical features to obtain insights into the material properties and functionality. Furthermore, the influence of the electrode layout as well as the activation conditions are studied aiming to provide a better understanding of the mechanism driving the electrochemical behavior of the electrodes. The EC activation enhances the electrochemical performance, provides a uniform electrochemical activity in the electrode's interface and allows the manipulation of the electrochemical properties of 3D printed electrodes by adjusting the duration of the treatment. CB/PLA electrodes offer a wide stable potential window that benefits their use in water‐based electrochemical applications. Thus, their suitability for Zn‐ion batteries and electrochemical sensing is explored, followed by their application in hydroquinone determination in water samples.

## Graphical abstract

Driven by the increasing need for sustainability this study proposes a simple and eco‐compatible activation method for carbon black – polylactic acid (CB/PLA) 3D printed electrodes. This method unlocks favorable features for the implementation of the electrodes in Zn‐ion energy storage and electrochemical sensing systems.

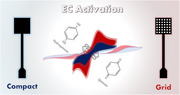

## 1 Introduction

Three‐dimentional (3D) printing is an emerging technology that allows the swift customization and fabrication of objects and devices to meet specific application necessities.^[^ ^1^, ^2^, ^3^, ^4^ ^]^ The sustainable and cost‐effective nature of this process renders it a powerful tool for small‐scale production of complex 3D structures on‐demand with just a desktop‐size printing machine, allowing its utilization across industrial, laboratory, and household settings.^[^ ^5^, ^6^, ^7^ ^]^

One of the most common 3D printing techniques is fused deposition modeling (FDM). This method involves heating and extruding filaments of thermoplastic materials such as polylactic acid (PLA) and acrylonitrile butadiene styrene (ABS) through a nozzle, layer‐by‐layer, forming 3D structures.^[^ ^8^ ^]^

The integration of carbon allotropes (graphene, carbon black, carbon nanotubes, etc.) in thermoplastic materials has facilitated the fabrication of conductive printable materials and significantly contributed to the widespread adoption of 3D printing technology across multiple and diverse fields such as sensing,^[^ ^9^, ^10^ ^]^ electronics,^[^ ^11^ ^]^ energy storage,^[^ ^12^, ^13^ ^]^ biomedical engineering,^[^ ^14^ ^]^ and robotics,^[^ ^15^ ^]^ producing low‐cost, in‐house devices.

The conductive filaments must contain a sufficient amount of carbon material that enables the formation of a continuous conductive path within the volume of the filament according to percolation theory^[^ ^16^ ^]^ and concurrently not surpassing the critical amount that will impact flowability and thermomechanical properties in a way that will affect the printability of the filament.^[^ ^17^ ^]^

In the field of electrochemistry, conductive filaments can be used as electrodes for energy and sensing applications, promoting the development of novel technologies with advanced features. However, most of the commercial conductive filaments available are not designed for electrochemical applications and lack beneficial characteristics for good electrochemical activity, such as high electrical conductivity, increased exposed conductive surface area, high charge transfer kinetics, good chemical stability and compatibility with electrolytes, especially due to the presence of intrinsic impurities. A pretreatment/activation step can enhance the electrochemical behavior of conductive filaments, unlocking their capabilities to form 3D printed electrodes with improved performance in various electrochemical applications.^[^ ^18^ ^]^

So far, various strategies have been employed to activate 3D printed electrodes. Browne et al. reported on the activation of graphene/PLA 3D printed electrodes with a combination of chemical and electrochemical method by immersing the electrodes in dimethylformamide (DMF) for 10 min and subsequently applying +2.5 V for 150 s in a phosphate buffer saline (PBS) as an electrolyte. This method enhanced the charge transfer characteristics and improved the performance toward electrocatalytic hydrogen evolution reaction.^[^ ^19^ ^]^ Later on, Santos et al. investigated the effect of electrochemical activation in graphene/PLA 3D printed electrodes, by applying positive potential followed by a voltammetric cycle toward negative potential values. Results indicated that the combination of positive and negative potential values granted beneficial charge transfer characteristics towards [Ru(NH_3_)_6_]Cl_3_, while the exposure of graphene sheets induced by the treatment offered promising sensing capabilities toward dopamine determination.^[^ ^20^ ^]^

Kalinke et al. utilized a combination of chemical and electrochemical activation in graphene/PLA 3D printed electrodes by immersing them in 1.0 M NaOH (for 30 min) or DMF (for 10 min) followed by +1.8 V for 900 s in phosphate buffer saline. As a result, the electrochemical (EC) activation leads to an exceptional heterogeneous electron transfer constant (k°obs) in comparison with the other activation methods; however, the combination of NaOH and EC activation was the optimum for dopamine determination.^[^ ^21^ ^]^ Gusmao et al. investigated the effect of (sono)chemical activation methods in graphene/PLA 3D printed electrodes by immersing them in protic and aprotic solvents; aprotic solvents performed better overall than the protic ones, while particularly DMF treatment offers better k°obs and better supercapacitor performance.^[^ ^22^ ^]^

In the case of CB/PLA, Ryl and coworkers reported on EC treatment and enzymatic hydrolysis as separate cases and explored the synergistic effect between the two methods. EC treatment with anodic polarization potentials can efficiently activate the 3D printed electrodes under both acidic and alkaline conditions, significantly enhancing charge transfer kinetics. The sole action of the studied electrolyte in the absence of the polarization component showed that alkaline environment is most effective in the PLA hydrolysis. On the other hand, enzymatic hydrolysis by proteinase K was slightly inferior to the EC treatment but its prolonged duration hinders its wide implementation. The combination of the EC treatment and enzymatic hydrolysis allowed a synergistic interaction for a more time‐efficient protocol.^[^ ^23^ ^]^ Munoz and coworkers mechanically polished the electrodes prior to EC activation in 0.5 M NaOH by applying +1.4 V for 200 s followed by −1.0 V for 200 s, the activated electrodes tested for the electrocatalytic activity over various analytes and used for the determination of dopamine,^[^ ^24^ ^]^ the simultaneous quantification of Cd^2+^ and Pb^2+ [^ ^25^ ^]^ and the analysis of hydroxychloroquine,^[^ ^26^ ^]^ exhibiting enhanced performance and promising sensing features.

The selection of the activation method of 3D printed electrodes is highly dependent on the electrode material and the intended application, as well as the shape and geometry of the electrodes.^[^ ^27^ ^]^ Each application has its own unique set of requirements, and some even have characteristics that contradict one another. Therefore, in order to promote one application over the other, it is critical to choose the most appropriate activation method.

Aqueous electrolytes are commonly used in electrochemical applications thanks to their non‐toxic nature, affordability, high dielectric constant, ionic conductivity, and abundance of water. Nevertheless, their use is limited by the relatively narrow electrochemical stability window due to the occurrence of electrocatalytic water‐splitting reaction.^[^ ^28^, ^29^ ^]^ As a result, the formation of gaseous bubbles and the generation of high currents at specific potential values can cause significant disruptions in the implementation of electrodes in electrochemical applications^[^ ^30^ ^]^ that take place in aqueous‐based electrolytes such as Zn‐ion batteries^[^ ^31^, ^32^ ^]^ and sensing^[^ ^33^ ^]^ applications.

In terms of the batteries, the continuous gas evolution not only lowers coulombic efficiency and affects cycling stability but also causes serious safety concerns deriving from the explosive nature of hydrogen gas in conjunction with the sealed unit character of rechargeable batteries.^[^ ^34^ ^]^

In what concerns the sensing applications, the generated bubbles may impede the analyte from reaching and attaching to the electrode's surface,^[^ ^35^, ^36^ ^]^ compromising the electrode sensing features. At the same time, the elevated currents produced during the water electrolysis can adversely affect the signal originating from the analyte, especially when it is located in regions of high potential values.^[^ ^37^ ^]^

In light of the above, it is of great importance to design new strategies and novel methodologies that can effectively mitigate the water‐splitting reaction, expanding the stable potential window. Such an approach would be highly beneficial for the development of enhanced sensing and energy storage applications.^[^ ^30^, ^38^ ^]^ These two types of applications draw from the same scientific field (electrochemistry), yet they have distinct and often conflicting requirements, while also have the potential for significant synergy. Materials and technologies that can function in these two fields can seamlessly contribute in the development of novel advanced applications, such as self‐powered wearable sensing devices.

Choosing the activation method for 3D printed electrodes is a complex process that requires careful consideration of the abovementioned parameters. In most studies, researchers rely on previous literature, and they rarely explore how the activation method or the activation parameters affect the target application. Therefore, it is important to personalize the activation method based on the specific characteristics of the electrode and the intended use.

**Scheme 1:**
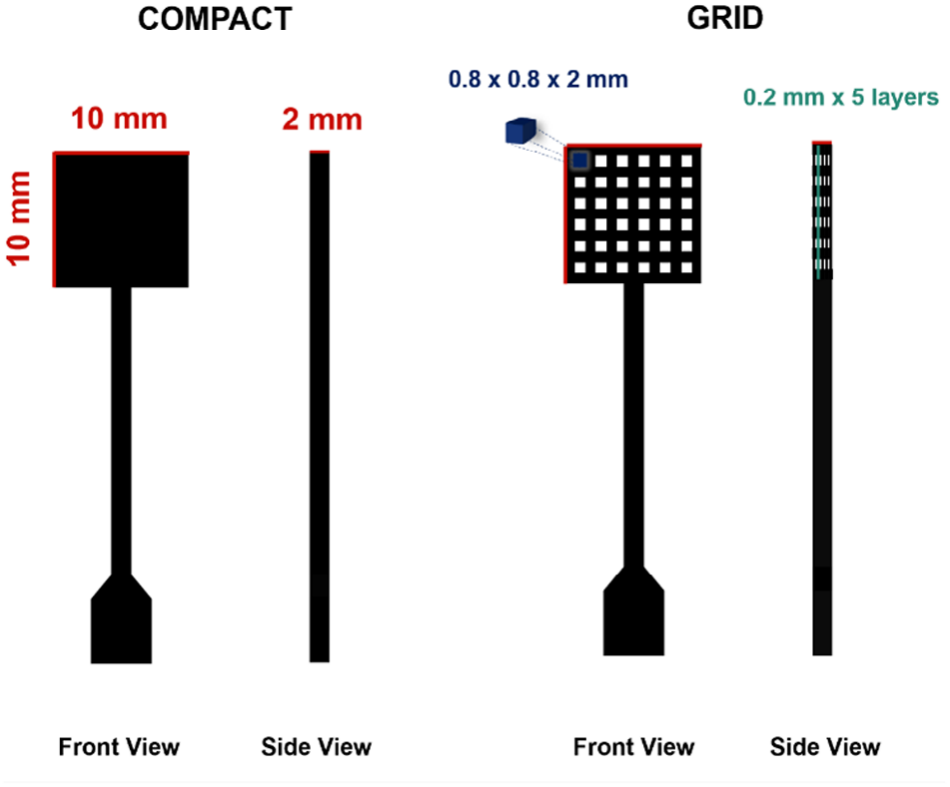
Graphical illustration of geometrical characteristics of compact and grid CB/PLA 3D printed electrodes.

Herein, we explore the applicability of CB/PLA 3D printed electrodes in electrochemistry, aiming to evaluate their performance and suitability in diverse electrochemical applications. EC treatment is employed as an activation method for CB/PLA 3D printed electrodes and the critical parameters that determine the electrochemical behavior of CB/PLA 3D printed electrodes. The layout of the electrode (whether it's compact or grid) (**Scheme** **1**), and the activation duration are meticulously analyzed to gain deeper insights into their impact on electrochemical performance. To this end, the CB/PLA 3D printed electrodes are characterized i) electrochemically by means of cyclic voltammetry (CV), electrochemical impedance spectroscopy (EIS), and scanning electrochemical microscopy (SECM), ii) structurally and morphologically via Raman spectroscopy, Fourier transform infrared spectroscopy (FTIR), scanning electron microscopy (SEM) and X‐ray photoelectron spectroscopy (XPS). Finally, the stable potential window of grid and compact CB/PLA rectangular 3D printed electrodes was evaluated prior their utilization as i) cathode at aqueous Zn‐ion energy storage systems and ii) sensing platforms for voltammetric analysis of various analytes, i.e., ascorbic acid, 1,3,5‐trinitrobenzene, hydroquinone, epinephrine (also known as adrenaline) and serotonin. Lastly, the performance of the electrodes is investigated over the amperometric determination of hydroquinone in tap water samples.

## 2 Results and Discussion

### 2.1 Geometrical Characteristics and Mechanical Behavior of the Electrodes

The CB/PLA electrodes were 3D‐printed with a thickness of 2 mm and a rectangular active surface area of 10 × 10 mm with two different layouts: compact and grid. In the compact layout the electrode material occupies the whole area of the electrode resulting in a dense and solid structure. The grid layout incorporates a pattern of evenly distributed cubical voids with dimensions of 0.8 × 0.8 × 2 mm. These voids are formed by 5 cross‐sectional layers, each 0.2 mm thick, that are equally distributed throughout the 2 mm thickness of the electrodes. A graphical illustration of the geometric characteristics of the CB/PLA 3D printed electrodes is shown in Scheme 1, and a photo of the electrode is presented in Figure S1A (Supporting Information).

Aiming to evaluate the mechanical behavior of the CB/PLA electrodes, a compression test was conducted on the rectangular active surface area of the grid and compact CB/PLA 3D printed electrodes, along with matching printouts crafted from plain PLA. The obtained stress strain curves are presented in Figure S1B (Supporting Information). The Young's modulus was calculated from the slope of the elastic deformation region as 1.12 GPa for plain PLA and 0.98 GPa for CB/PLA indicating that the integration of carbon black in the thermoplastic material decreases the stiffness of the filament and leads to more flexible printouts. The decreased failure strain of the compact CB/PLA is similar to the plain PLA printouts denoting a more stiff and brittle nature of the electrode in comparison to the grid CB/PLA electrode. Besides Young's modulus, which is an intrinsic property of the material, the structural differences affect the stiffness and the overall mechanical behavior of the electrodes.

### 2.2 Activation Method Selection

The electrochemical applications and the activation treatment protocols aiming to improve the electrical properties of conductive filaments have attracted a lot of interest.^[^ ^39^, ^40^ ^]^ In the quest to enhance the conductivity and charge transfer kinetics of 3D printed electrodes, various activation methods have been proposed. These methods include solvent treatment (chemical method),^[^ ^41^ ^]^ sonochemical treatment,^[^ ^22^ ^]^ electrochemical treatment,^[^ ^26^ ^]^ laser ablation,^[^ ^42^ ^]^ mechanical treatment,^[^ ^43^ ^]^ spark discharge activation,^[^ ^44^ ^]^ and others. The impact of one or a combination of these methods on 3D printed electrodes depends on the filament material and the applied conditions. The successful implementation of an activation protocol over one type of filament does not guarantee a similar performance over the other.^[^ ^41^ ^]^ Table S1 (Supporting Information) summarizes the features of recently proposed activation methods and protocols based on the filament material to which they were applied.

In this work, the primary selection of activation methods and applied parameters is based on methods applied for graphene/PLA^[^ ^21^ ^]^ 3D printed electrodes but rarely used under these conditions for CB/PLA. Taking into consideration parameters such as sustainability, ease of use and time efficiency, the methods under investigation are i) EC treatment in PBS at +1.8 V for 900 s, ii) chemical treatment by immersing the electrode in 1.0 M NaOH for 30 min (saponification) and iii) the combination of them by immersing the electrode in NaOH for 30 min and then applying +1.8 V for 900 s in PBS (NaOH/EC).

Cyclic voltammograms (CVs) in the presence of 0.5 mM hexacyanoferrate (III), of compact and grid rectangular 3D printed CB/PLA electrodes are demonstrated in Figure S2 (Supporting Information). All activation methods led to improved electrochemical behavior over the hexacyanoferrate redox transition compared to the as‐printed electrodes. In particular, the electrodes involving an EC activation step had lower peak‐to‐peak separation (ΔEp) with a value of ≈0.5 V against the non EC activated that is ≈0.8 V, pointing toward its promising role as a pretreatment method. At the same time, the two different layouts of electrodes (compact and grid) displayed analogous trends, albeit to varying degrees.

**Figure 1:**
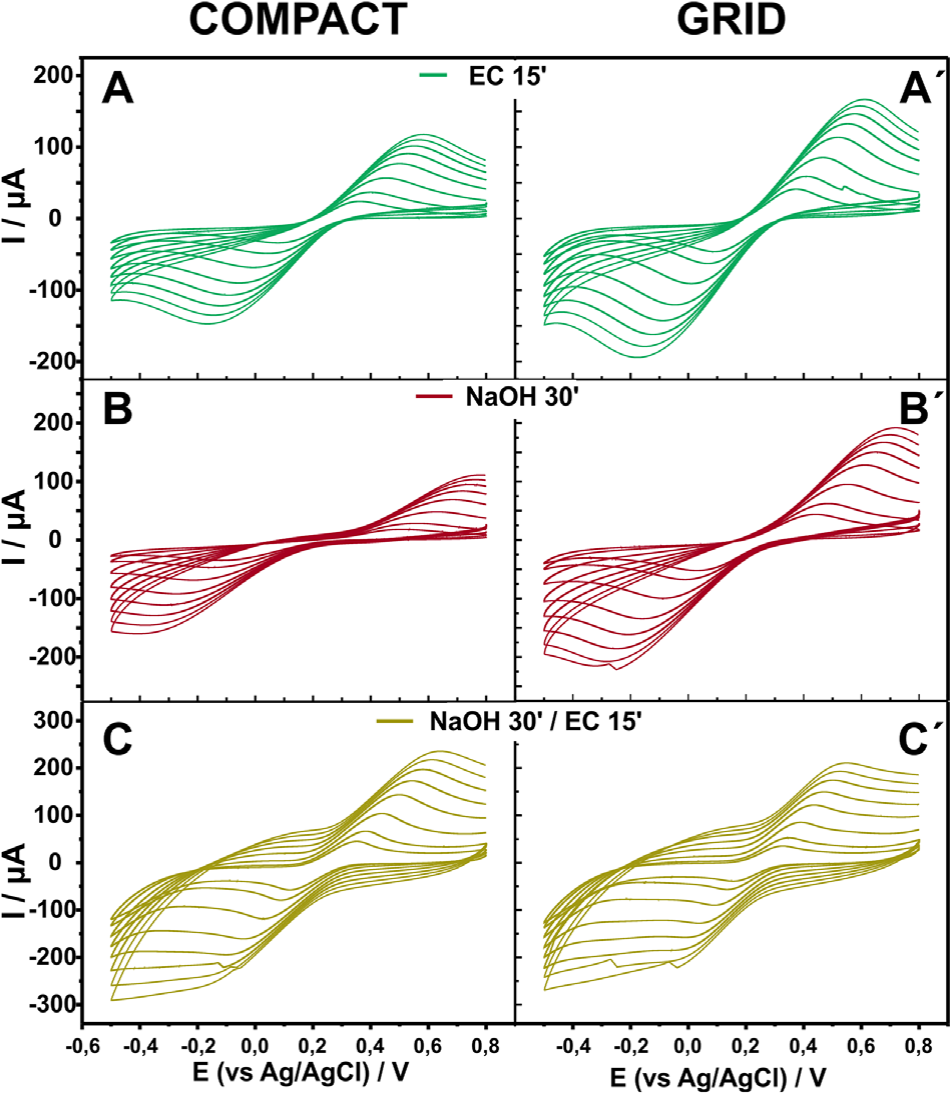
CVs of compact (A–C) and grid (A′‐C′) CB/PLA 3D printed electrodes at a scan rate from 5–150 mV s^−1^ in 0.1 M PBS (pH 7) containing 0.5 mM hexacyanoferrate (III), after the (A‐A′) EC activation for 15 min, (B‐B′) saponification for 30 min, and (C─C′) combination of saponification and EC activation.

Considering that, selecting method of activation relying on just a single scan rate CV is challenging and may result in deceptive decisions. Therefore, we chose to compare the activation methods by evaluating the performance of the electrodes over a range of scan rates. **Figure** **1** demonstrates the CVs of compact and grid 3D printed electrodes in the presence of 0.5 mM hexacyanoferrate (III) over the scan rate of 5–150 mV s^−1^. All the activation methods exhibit nice and well‐formed CVs in low scan rates, while the increase of the scan rate reveals poor electrochemical behavior in the activation methods involving saponification through immersing into NaOH.

Saponification alone increases ΔΕp, indicating poor electrocatalytic activity of 3D printed electrodes. Upon the combination with EC treatment, the ΔΕp is improved by nearly 400 mV, surpassing slightly the one resulting just from the EC treatment. Despite this, the increase in the scan rate reveals an increased capacitive current. In that case, the faradaic current attributed to the hexacyanoferrate redox transition is superimposed on the capacitive current. Hence, although the overall current appears higher in the NaOH/EC method, the faradaic current is not superior to the EC method alone.

In what concerns the comparison between the two electrode layouts, they perform similarly in case of EC activation, with almost the same ΔEp and a slightly higher peak current (Ip) in the grid electrode. However, activation methods that involve saponification seem to be more effective in improving the ΔΕp of grid electrodes compared to compact electrodes due to the increased surface area that leads to an expanded count of carbon black active sites upon activation.

Therefore, taking into consideration both the ΔΕp and the faradaic Ip, the EC method facilitates the electrochemical behavior of the electrode even for a smaller duration of treatment, and to a similar extent over different electrode layouts. Hence, EC is deemed the optimal method to proceed studying the electrochemical behavior of the CB/PLA 3D printed electrodes.

Apart from the electrochemical performance, the activation methods have strengths and weaknesses that depend on their nature and the experimental conditions (Table S1, Supporting Information). Methods that involve chemical treatment prevail in terms of scalability, allowing mass activation of 3D printed electrodes, but lack in terms of sustainability due to the toxic chemicals and hazardous wastes that can contaminate soil and water sources, posing a threat to public health and safety. Mechanical methods cause surface inhomogeneities and lead to questionable reproducibility. However, this method is noteworthy as the only one that provides reusability to the 3D‐printed electrodes.

While some of the reported methods may achieve higher electrochemical performance, the selected EC method stands out for its sustainable and eco‐compatible character. As it is a single‐step method in an aqueous‐based electrolyte (i.e., 0.1 M PBS pH 7) that offers excellent balance of effectiveness, reproducibility, simplicity, and sustainability. This method is suitable for applications requiring rapid and straightforward electrode activation while it concurrently avoids the use of extreme conditions or complex processes and offers mechanical and chemical integrity that will benefit the stability of the 3D printed electrodes for long‐lasting applications such as batteries.

### 2.3 Electrochemical Characterization

Aiming to optimize the EC activation parameters and explore their impact on the electrochemical behavior of the CB/PLA 3D printed electrodes, CVs in the presence of hexacyanoferrate (III) 0.5 mM were measured at a scan rate of 25 mV s^−1^. Figure S3 (Supporting Information) displays that only anodic potential is able to activate CB/PLA 3D printed electrodes while using cathodic potential has no benefits. Additionally, +1.8 V is the minimum potential value that enhances electrochemical performance of the electrodes, while slightly higher potential values have negligible impact on the ΔΕp and Ip of hexacyanoferrate (III).

**Figure 2:**
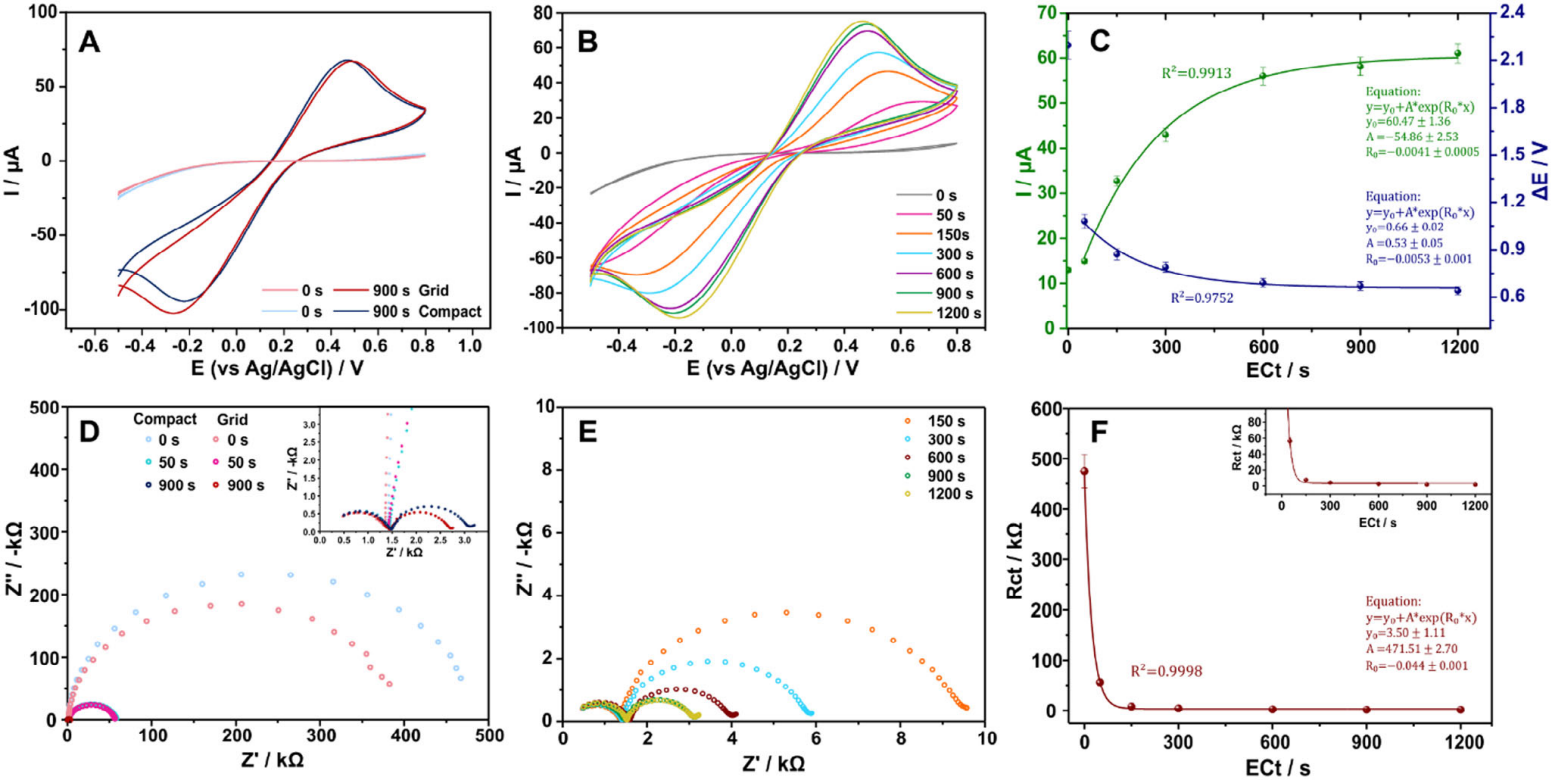
CVs of CB/PLA 3D printed electrodes in 0.1 M PBS (pH 7) containing 0.5 mM hexacyanoferrate (III) at a scan rate of 25 mV s^−1^, A) for compact and grid type of electrodes before and after activation for 900 s and B) for different activation time intervals from 0 s to 1200 s of compact electrode along with C) the corresponding correlation plot of activation time (*EC*_t_) with ΔΕp and Ip. Nyquist plots of compact and grid 3D printed electrodes in 0.1 M PBS (pH 7) containing 0.5 + 0.5 mM hexacyanoferrate (III)/(II), D) for compact and grid electrodes before and after activation for 50 s and 900 s (inset graph) and E) for different activation time intervals from 150 to 1200 s of compact electrode along with F) the corresponding correlation plot of activation time with *R*_ct_.

Electrochemical behavior of compact and grid electrodes before and after EC treatment is displayed in **Figure** **2**A, where both types of electrodes exhibit a superior electrochemical behavior after the EC treatment denoted by the concomitant enhancement of ΔΕp and Ip of the hexacyanoferrate (III).

In what concerns the activation duration, the electrochemical behavior of the CB/PLA electrodes improves progressively as the activation duration (ECt) increases which is reflected through the decrease of ΔEp and increase in the Ip (Figure 2B). However, the improvement reaches a plateau at ≈600 s, as further prolongation in activation time has a small‐to‐negligible effect. Data reveal exponential correlation between ΔEp and ECt and between Ip and ECt with the correlation coefficient R^2^ of 0.9752 and 0.9913, respectively (Figure 2C). Interestingly, just 50 s of EC treatment reduced the ΔΕp in half value while the Ip was minimally impacted. This suggests that the increased electrocatalytic activity is a result of the initial exposure of CB caused by the effect of the anodic potential, while prolonged EC activation, increased the electroactive surface area and charge transfer kinetics, leading to higher Ip as dictated by the modified Randles–Sevcik equation that applies in systems with nΔΕp > 200 mV that is $I_{p}=2.99\times 10^{5}\;\sqrt{\alpha }A\sqrt{D}C\sqrt{v}$ where Ip is the forward peak current, α is the transfer coefficient calculated through the equation $|E_{p}-E_{p/2}|=\frac{47.7}{\alpha }$ (at 25 °C), A is the electroactive surface area, D is the diffusion coefficient, C the concentration of the redox species in the solution and ν is the potential scan rate.^[^ ^45^ ^]^

EIS spectra in the form of Nyquist plots in the presence of 0.5 + 0.5 mM hexacyanoferrate (III)/(II) in 0.1 M PBS pH 7, are demonstrated in Figure 2D,E. All the Nyquist plots feature two semi‐circles indicative of two distinct electrochemical processes occurring on the electrodes with different time constants. The semicircle observed at higher frequencies is unaffected by the layout of the electrode and the activation treatment and does not take part in any charge transfer reaction. In contrast, the diameter of the second semicircle, which varies with *EC*_t_, represents the charge transfer resistance (*R*_ct_), highlighting the impact of activation on electrode conductivity.^[^ ^46^ ^]^

A comparison between the compact and grid electrodes before and after EC activation is shown in Figure 2D, where the non‐activated electrodes exhibit enormous value of Rct. EC activation improves the electrochemical behavior of the electrodes, decreasing the *R*_ct_ from 475 and 382 kΩ to 56 and 54 kΩ (for 50 s) and eventually to 1.6 and 1.2 kΩ (for 900 s) for compact and grid electrodes, respectively. Interestingly, the *R*_ct_ difference between the two electrode types was significantly higher before EC activation, as compared to the *R*_ct_ difference after activation. This indicates that EC activation not only improved the electrochemical behavior of the electrodes but also almost evened out the *R*_ct_ of the two different electrode layouts.

Further analysis of the effect of *^E^*_Ct_ on impedance of the electrode (Figure 2E) reveals an incremental decrease of *R*_ct_ with increasing *EC*_t_, reaching a plateau at 900 s. Beyond this point, additional activation time does not further enhance electrochemical performance. Up to the point of plateau, data reveal an exponential correlation between *R*_ct_ and *EC*_t_, with the correlation coefficient R^2^ being 0.9998 (Figure 2F).

The dependance of the electrochemical properties from the *EC*_t_ enables the control of the electrochemical behavior of the CB/PLA electrode rendering it a useful tool for manipulating the properties of an electrochemical system as per demand following the requirements of the intended application simply by adjusting the activation duration.

**Figure 3:**
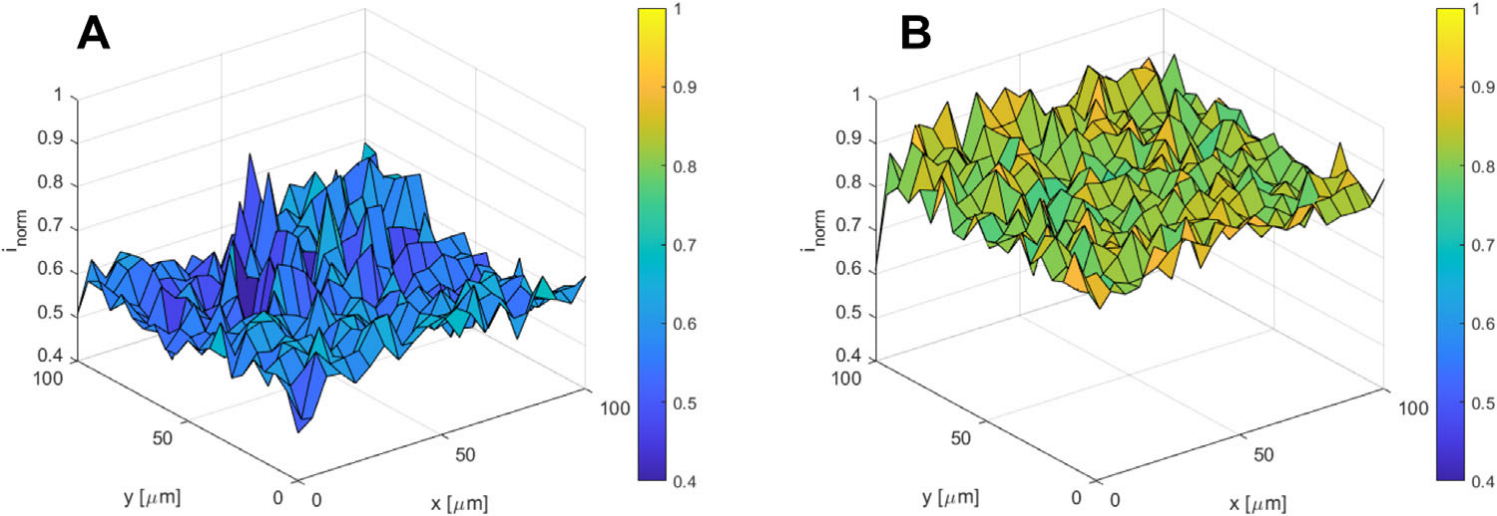
SECM 3D maps of the A) non‐activated and B) EC activated at +1.8 V for 900 s CB/PLA 3D printed electrodes displaying the faradaic current of hexacyanoferrate (II) redox transition measured in an area of 100 µm^2^ of the compact electrodes.

SECM was utilized aiming to investigate the localized electrochemical activity of CB/PLA 3D printed electrodes upon EC activation. Hence, **Figure** **3** illustrates the mapping of 100 µm^2^ of the non‐activated and EC activated compact electrodes through the measurement of Faradaic current of 10 mM hexacyanoferrate (II) redox transition on the surface of the 3D printed electrodes with a 10 µm ultramicroelectrode in a 4D‐array scan (i.e., point by point with an increment of 5 µm). Data display that EC activation not only improves electrode's electrochemical activity but also results in homogeneous electrochemical behavior across its surface, as indicated by the small discrepancies in the current values, unlike the non‐activated electrode.

### 2.4 Structural and Morphological Characterization

Raman spectroscopy was used to obtain insights on the CB/PLA 3D printed electrodes concerning structural properties, defects, and hybridization changes that occurred during EC activation. As shown in Figure S4A (Supporting Information), two characteristic bands of carbon‐based materials were observed. The first one is the D band (1400 cm^−1^), which is associated with sp^3^ hybridization and the presence of defects while the second one is the G band (1600 cm^−1^), which is correlated with the presence of sp^2^ carbon. The location of the two bands and the intensity ratio I_D_/I_G_ remained unchanged during the EC activation, with a value of 0.87. This indicates that the EC activation did not introduce any structural defects to the electrodes. These results are consistent with previous Raman studies of EC activated CB/PLA 3D printed electrodes.^[^ ^47^, ^48^ ^]^

FTIR analysis was utilized for the characterization of the surface functional groups. Figure S4B (Supporting Information) display the FTIR transmission spectra of plain PLA and conductive filaments (CB/PLA) before and after EC activation. The plain PLA filament and CB/PLA filament both exhibit similar bands associated with the PLA polymer, despite differing in intensity. In particular, the peak at 1740 cm^−1^ is designated to the stretching vibration of C═O, the peaks at 1464, 1182, and 1076 cm^−1^ are associated with the CH_3_ functional group, specifically 1464 cm^−1^ corresponds to the C─H bending vibration, 1182 cm^−1^ to the CH_3_ rocking vibration, and 1076 cm^−1^ to the C─CH_3_ stretching vibration. Transmission band at 870 cm^−1^ associated with C─COO stretching is apparent only in plain PLA, while CB/PLA in general exhibit much lower absorption intensity.^[^ ^49^, ^50^ ^]^ In what concerns the activation process, it seems that the FTIR spectra are identical with and without any EC treatment.

**Figure 4:**
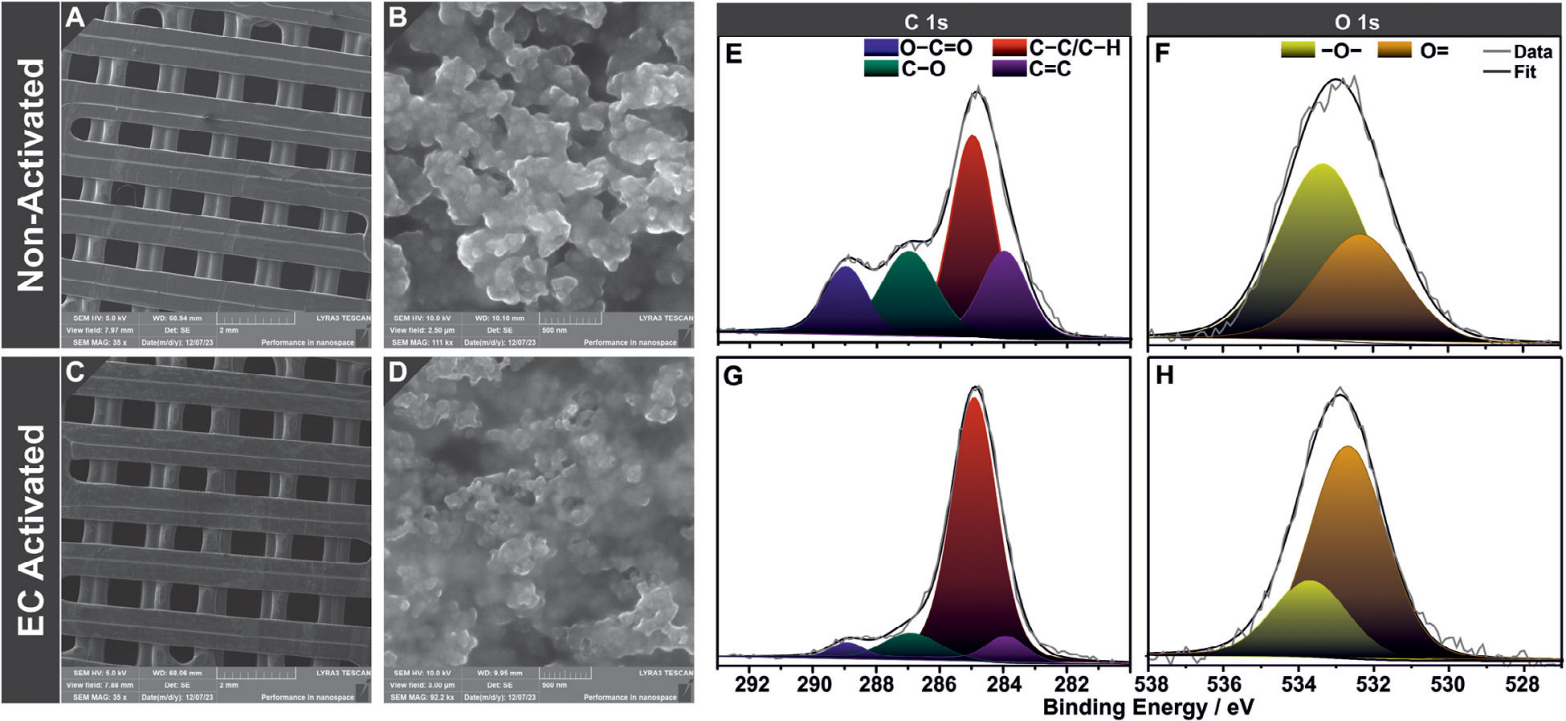
A‐D) SEM images and high resolution XPS spectra of E,G) C 1s and F,H) O 1s of grid‐layout CB/PLA 3D printed electrodes before (A,B,E,F) and after (C,D,G,H) EC activation at +1.8 V for 900 s in 0.1 M PBS (pH 7).

SEM images in **Figure** **4** depict grid layout CB/PLA 3D printed electrodes before (Figure 4A,B) and after EC activation at +1.8 V for 900 s (Figure 4C,D). Low magnification images provide an accurate portrayal of the grid layout of the electrodes and their smooth surface, confirming minor discrepancies between the designed (Scheme 1) and the printed (Figure 4A,C) models. At the same time, the higher magnification reveals a sponge‐like ingrained morphology of the CB agglomerates in the PLA that constitute the 3D printed electrodes. The comparison between the SEM images suggests small differences in morphology between the non‐activated and EC activated electrodes, mostly on the size of the agglomerates, in which the non‐activated is slightly bigger.

The surface composition of 3D printed CB/PLA electrodes and the changes upon EC activation are conducted through XPS analysis. The survey spectra of non‐activated and EC activated electrodes shown in Figure S5 (Supporting Information) display a difference in the ratio of C and O caused by the EC treatment. Figure 4E–H display high‐resolution (HR) core‐level spectra for C 1s and O 1s, providing a detailed overview of the changes occurring at the electrode interface. C signals are synthesized by four components attributed to C═C (284 eV), C─C/C─H (285 eV), C─O (287 eV) and O─C═O (289 eV), where the C═C component originates from the CB. Activation seems to reduce the peaks attributed to O─C═O and C─O from PLA functional groups, likely due to its dissolution during the activation. This is confirmed by the shape of C 1s region spectrum, which is consistent with the XPS of PLA^[^ ^51^ ^]^ prior to activation. The increase in C─C/C─H component accompanied by a decrease in C═C concentration was also previously reported for and attributed to the oxidation of sp^2^ hybridized carbon.^[^ ^52^ ^]^ On the other hand, O signals are synthesized by two components attributed to ─O─ (533 eV) and O = (532 eV), whose ratio changes due to EC treatment. XPS analysis provides a better understanding over the poor electrochemical behavior of the non‐treated 3D printed electrodes and the mechanism of action of EC treatment to improve these electrochemical properties by exposing the CB in the electrode interface.^[^ ^23^ ^]^

Structural and morphological characterization of CB/PLA electrodes revealed that the EC activation process is mild in nature and does not affect the electrode's structural and morphological characteristics, but only the surface composition. In contrast with other activation processes that involve hazardous organic solvents.^[^ ^53^, ^54^ ^]^

### 2.5 Stability of the Potential Window

Electrochemical applications in aqueous electrolytes hold multiple benefits in terms of cost and sustainability. The abundance, nonflammable nature, and overall safety they provide, in combination with good ionic conductivity and the high dielectric constant, render aqueous electrolytes prominent in battery technology. Nevertheless, there are some limitations to the full exploitation of such electrolytes in batteries. For instance, water has a narrow voltage stability window, as it can be reduced, releasing H_2_ gas through hydrogen evolution reaction (HER) at negative potentials or oxidized releasing O_2_ gas through oxygen evolution reaction (OER) at positive potentials. The combination of these reactions is known as electrocatalytic water‐splitting reaction. It is considered one of the best ways to produce both hydrogen and oxygen. Still, yet in energy storage applications, they could lower the coulombic efficiency, affect cyclic stability, and cause serious safety concerns while in sensing, it can adversely affect i) the analyte capability to reach the electrode's interface due to the bubble production and ii) the signal readability due to increased catalytic currents.

Therefore, there is a growing demand for electrode materials that can benefit the performance of water‐based electrochemical applications by suppressing the electrocatalytic water‐splitting reaction following an anticatalytic strategy.^[^ ^30^ ^]^ This will provide a wide stable polarization window that will facilitate sensing and energy storage applications.

In order to test the suitability of the CB/PLA 3D printed electrodes for aqueous‐based Zn‐ion batteries, the electrodes are tested both for their electrocatalytic activity toward HER and OER (Figure S6, Supporting Information) and the intercalation/deintercalation of Zn through cyclic voltammetry (Figure S7, Supporting Information). Figure S6A,B (Supporting Information) depicts the linear sweep voltammetry (LSV) polarization curves for the 3D printed electrodes before and after the EC activation under neutral conditions. As expected, activated electrodes perform better than non‐activated, but both of them display limited electrocatalytic activity, leading to a wider stable potential window. This behavior is deemed promising for use in aqueous‐based batteries, avoiding gas evolution during Zn cycling and offering exceptional stability (Figure S8, Supporting Information).

Further discussion and analysis of the Zn electrochemistry of CB/PLA 3D printed electrodes can be found in the Supporting Information.

### 2.6 Sensing Applicability

The sensing applicability of grid and compact CB/PLA 3D printed electrodes was investigated by assessing their electrocatalytic activity for 10 µM of selected electroactive species in 0.1 M PBS pH 7. A range of analytes were selected based on their diverse electrochemical behavior and significance in chemical analysis, aiming to gain a comprehensive understanding of the sensing properties of the 3D printed electrodes. Hence, the substances tested own diverse redox properties and, at the same time, are highly sought‐after analytes in fields of medicinal, environmental, and food chemistry.

Ascorbic acid, well‐known as vitamin C is a vital nutrient that is present in many foods and drinks and is essential for the health and proper functioning of living organisms.^[^ ^55^ ^]^ Serotonin and epinephrine are hormones excreted in the human body during stress and “dysfunction”; their abrupt increase is a sign of health risks, while their concentration can provide additional health information.^[^ ^56^, ^57^ ^]^ Hydroquinone and 1,3,5‐trinitrobenzene are emerging pollutants that require monitoring due to their high toxicity and/or explosive nature, which can adversely affect the ecosystem.^[^ ^58^, ^59^ ^]^

Besides their usefulness in chemical analysis, these analytes were selected as representative examples of electroactive species with diverse electrochemical behavior that will help unravel the potential of CB/PLA electrodes for the electrochemical analysis of them as well as other similarly behaving compounds.

**Figure 5:**
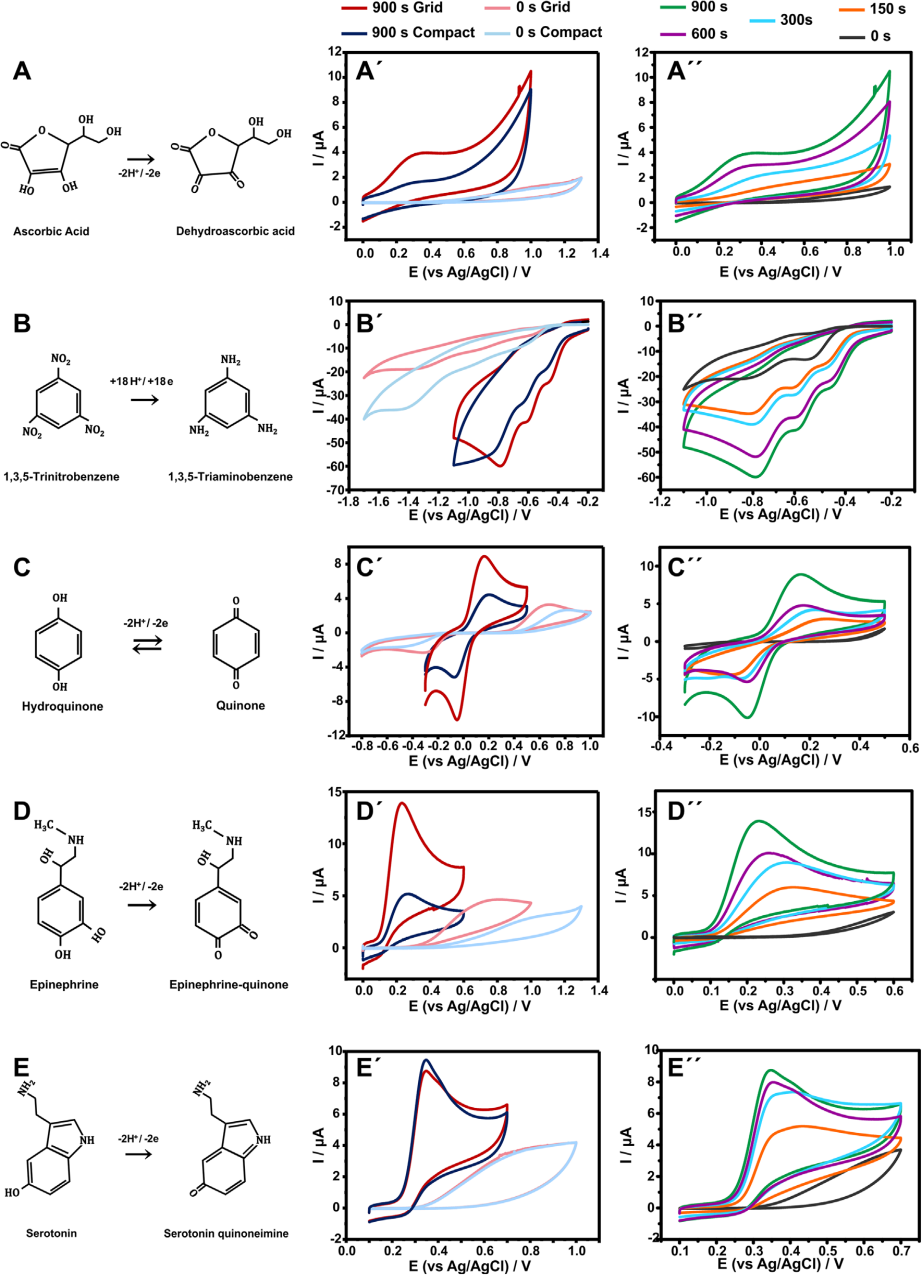
Electrocatalytic activity of CB/PLA 3D printed electrodes in the presence of 10 µM of A‐A″) ascorbic acid, B‐B″) 1,3,5‐trinitrobenzene, C─C″) hydroquinone, D‐D″) epinephrine and E‐E″) serotonin in 0.1 M PBS pH 7. Tentative reaction mechanisms describing the occurring electrochemical processes (A–E), CVs of EC activated (900 s) and non‐activated (0 s), compact and grid electrodes (A′‐E′), and CVs of grid electrodes activated for varying durations from 0 to 900 s (A″‐E″). Scan rate of 25 mV s^−1^.

**Figure** **5** depicts the electrochemical reaction taking place in the electrode interface (Figure 5A–E) along with the electrocatalytic behavior of grid and compact CB/PLA 3D printed electrodes before and after the EC activation at +1.8 V for 900 s (Figure 5A′–E′). Additionally, Figure 5A″–E″ depicts the performance of grid electrodes EC activated with different time intervals (150, 300, 600, 900 s).

Electrooxidation of ascorbic acid to dehydroascorbic acid is a reversible electrochemical electron transfer of an irreversible chemical reaction (Figure 5A), which overall leads to an irreversible process.^[^ ^60^ ^]^

On non‐activated electrodes, the electrooxidation of ascorbic acid occurs at a high potential of 1.03 V and is represented by small and flattened peaks. Activation of the electrodes lowers the oxidation potential to 0.31 V and significantly increases the peak current. This concurrent increase in the peak current and lowering in the peak potential by 720 mV denotes a great electrocatalytic activity of the EC activated electrodes over the electrooxidation of ascorbic acid. This behavior is attributed to the large surface area and hence the increased exposed active CB sites that can facilitate the reaction.

Intriguingly, the non‐activated electrodes demonstrate uniform performance, whereas the EC activation distinctly differentiates their performance, in terms of peak current.

In particular, the EC activation leads to comparable peak shifting in both layouts of electrodes, but the peak current response differs significantly, highlighting the effect of activation in electrodes of different layouts.

Furthermore, Figure 5A″ showcases a gradual shifting in the peak position to lower values with a concomitant increase in peak current, which underscores the role of *EC*_t_ in the sensing performance of CB/PLA 3D printed electrodes. Even 300 s of activation can enhance the performance by reducing the reaction overpotential (by 630 mV), but still, the peak current increases as the activation process prolongs and higher surface area becomes electrochemically active. These findings emphasize the importance of carefully adjusting the activation time when seeking to optimize the electrocatalytic performance of such electrodes.

The electrochemical reduction of 1,3,5‐trinitrobenzene occurs in three steps (Figure 5B), where each of the three nitro groups is reduced to its corresponding hydroxylamines and eventually, amines.^[^ ^61^, ^62^ ^]^ As a result, three distinct peaks are formed at the potentials of −0.60, −0.95, and −1.35 V when non‐activated electrodes are used. However, upon activation, the potential of these peaks shifts to −0.46, −0.61 and −0.81 V. The EC activation not only shifts the peaks to a lower potential but also brings the peaks closer to each other (Figure 5B′).

Similarly to ascorbic acid, a short activation time can shift the peaks close to their “final” position, while a further activation seems to have only a marginal effect on enhancing the peak current intensity (Figure 5B″).

Hydroquinone undergoes a redox process involving two‐electron transfer reaction, leading to the formation of quinone. The redox process can be visualized through a pair of peaks, an oxidation peak followed by a reduction peak (Figure 5C). The distance between these peaks is controlled by the type of electrode used and the activation parameters. When it comes to compact non‐activated electrodes, the redox peaks can be observed at +0.75 and −0.45 V, with a ΔΕp of 1.20 V. Meanwhile, grid electrodes will exhibit peaks at +0.64 and −0.27 V, with ΔE of 0.91 V (Figure 5C′).

The two types of electrodes differ in what concerns electrocatalytic activity as denoted by ΔΕp, but both of them exhibit an irreversible redox process with sluggish charge transfer kinetics before activation.

Upon EC activation, both types of electrodes demonstrate the redox peaks at +0.18 V and −0.06 V (ΔΕp = 0.24 V), turning the process into a quasi‐reversible. EC activation can improve the electrocatalytic properties of the electrodes, resulting in better charge transfer kinetics, and decreasing the ΔΕp. Even though the ΔΕp became identical, still the two electrode layouts differentiate in term of peak current, as the grid electrode outperforms the compact one.

In what concerns the effect of activation time, the ΔΕp changes gradually by increasing the activation time (Figure 5C″), similarly to the case of ascorbic acid. The most significant change occurs from 0 to 150 s, while smaller changes are observed by further increasing the activation duration. ΔΕp reached a plateau at 600 s, while peak current improved notably in 900 s, denoting that ΔΕp and peak current are not reaching their maximum at the same point, as the charge transfer kinetics enhance instantly while the electroactive surface area keeps increasing over time.

The oxidation process of epinephrine leading to its quinone‐derivative is represented by an anodic peak in the CV, as shown in Figure 5D.^[^ ^63^ ^]^ Prior activation, compact and grid electrodes exhibit different electrocatalytic activities, with grid electrodes showing a peak at a lower potential (+0.72 V) with a higher peak current than compact electrodes whose peak is located at +1.0 V (Figure 5D′). Activation results in a peak shifting at +0.24 V for both grid and compact electrode while the current difference between them increases. An ECt of 150 s effectively shifts the peak to +0.3 V, which is then followed by a gradual but noticeable peak current increase as the *EC*_t_ increases (Figure 5D).

Serotonin electrooxidation to serotonin quinoneimine (Figure 5E) on EC activated CB/PLA electrodes does not follow the same trends as the previously tested electroactive species (Figure 5E′). Besides the main oxidation peak, a second neighboring peak, attributed to adsorbed serotonin, is observed in both electrodes, prevailing in the grid electrode due to the numerous active sites allowing the adsorption of the analyte. This double peak could make the determination of serotonin difficult when using the peak height; alternatively, the overall charge that passes the cell can be used by addressing the integrated area from the onset potential.^[^ ^64^ ^]^ Compact and grid electrode demonstrate a different ratio of the two peaks but own the same integrated area, hence overall charge.

The performance of different activation times is affected by the adsorbed species even more (Figure 5E″). An activation procedure of 300 s demonstrates higher peak current in the secondary peak, and as the *EC*_t_ increases the main oxidation peak takes over. In contrast to previous examples, the electrocatalytic activity does not enhance much after 300 s of activation, as the bigger difference between 300, 600 and 900 s activated electrodes is the peaks' current ratio.

Overall, the EC activated CB/PLA 3D printed electrodes hold great promise as sensing platforms thanks to the simultaneous i) lowering of redox potential and ii) increase in peak current response in the presence of a concentration as low as 10 µM in all the tested analytes. It is worth noting that the sensing performance of compact and grid electrodes and the impact of EC activation on them differs from one analyte to another, dictating the necessity of investigating these parameters not only by studying electrochemical properties through commonly used electroactive species (such as hexacyanoferrate (III)), but also optimizing it over the intended application.

### 2.7 Optimization and Calibration Features

EC activation provides enhanced performance toward the electrochemical reactions of various analytes, holding promise for excellent analytical figures‐of‐merit. Aiming to explore in detail the performance and the applicability of the EC activated CB/PLA 3D printed electrodes, hydroquinone was selected as a model analyte and its amperometric determination was investigated.

First, the catalytic currents were recorded at various potentials ranging from +0.1 to +0.3 V (Figure S9, Supporting Information) over the consecutive additions of hydroquinone (3 × 2 µM, 3 × 5 µM). The I‐t curves demonstrated in Figure S9 (Supporting Information) show a linear current response with the hydroquinone concentration, which is evidence of the suitability of all the tested potential values as appropriate polarization potentials for the amperometric determination of hydroquinone. Nevertheless, the sensitivity of the method improves at higher potential values. Hence, +0.3 V has been chosen as the optimal polarization potential for further investigation.

**Figure 6:**
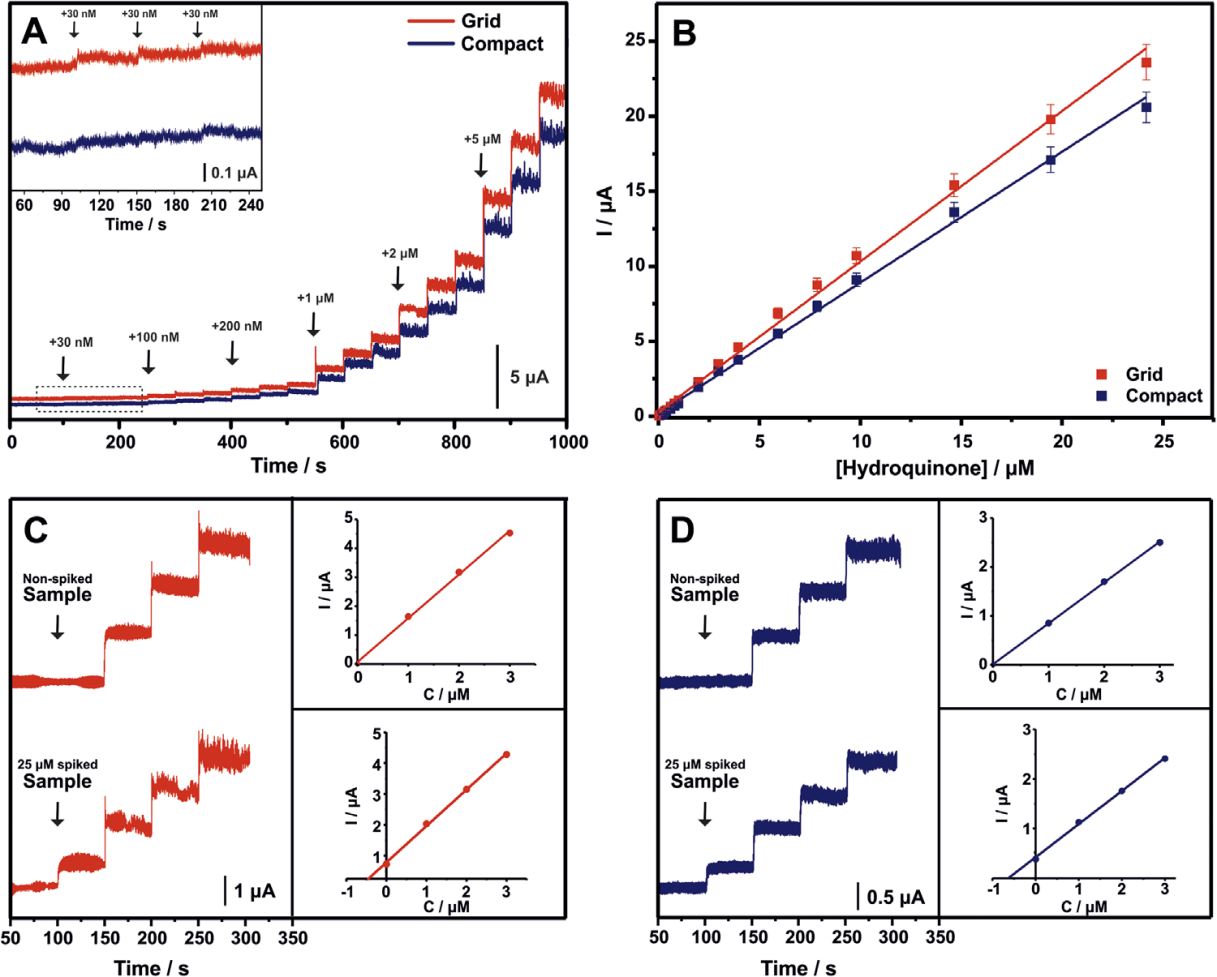
A) Amperometric curves recorded on EC activated CB/PLA grid (red) and compact (blue) 3D printed electrodes at polarization potential of +0.3 V over the concentration range from 30 nM to 24.2 µM and B) the respective calibration plots. The inset graph shows the response of the two electrodes at low concentrations on an enlarged scale. Amperometric curves along with the respective standard addition plots of the non‐spiked and the 25 µM spiked tap water samples measured on C) grid and D) compact 3D printed electrodes.

Under these conditions, the calibration features of grid and compact EC activated CB/PLA 3D printed electrodes for the amperometric determination of hydroquinone were evaluated. **Figure** **6**A depicts the chronoamperometric plots recorded in 0.1 M PBS (pH 7) at +0.3 V with triple addition of each of the following hydroquinone concentrations: 30, 100, 200, 1000, 2000, 5000 nM. The corresponding calibration plots (Figure 6B) demonstrate a linear correlation between the current response and hydroquinone concentration from 0.03 to 24.2 µM for the grid electrode and from 0.1 to 24.2 µM for the compact electrode. The data fit the regression equation *I* (µA) = 1.0039 × *C*(µ*M*) + 0.277 ( *R* ^2^ = 0.9969) for the grid and *I* (µA) = 0.873 × *C*(µ*M*) + 0.1811 ( *R* ^2^ = 0.9977) for the compact, with the LOD, calculated as 3σ/slope, to be 14.8 and 36.4 nM for grid and compact electrode, respectively.

Comparison of the analytical figures of merit of grid and compact EC activated CB/PLA 3D printed electrodes with other carbon‐based electrochemical sensors in **Table** **1** reveal that our electrodes outperform most of the reported methods for the determination of hydroquinone, even though they are bare electrodes without any electrocatalytic materials or modification applied on their surface. The low cost, on‐demand fabrication of the electrodes in combination with the environmentally friendly nature of the activation process make them robust and sustainable sensing platforms that hold promise for improved analytical performances over a wide range of analytes.

**Table 1:** Comparison of analytical figures of merit of carbon‐based electrochemical sensors for the determination of hydroquinone.

Electrode	Technique	LOD [µM]	Linear Range [µΜ]	Ref.
ECF‐CPE	DPV	0.4	1–200	[65]
GMC/GCE	DPV	0.37	2–50	[66]
NCNT@CNFs/GC	DPV	0.05	0.1–425	[67]
KOH‐activated GSEC film	SWV	0.1	0.5–200	[68]
3D‐OPPy/ERGO/GCE	DPV	0.1	0.5–100	[69]
TCpSi/GCE	DPV	1.4	4.6–35.2	[70]
SA‐900/GCE	SWV	0.018	0.05–1.5	[71]
CB‐PLA	DPV	0.22	1–50	[72]
HC/GCE	Amperometry	1.05	1–3000	[73]
LC/GCE	Amperometry	0.095	0.5–1000	
Graphite‐sparked KB/SPE	DPV	0.002	0.05–1	[74]
CB/PLA 3DE (Compact) – EC	Amperometry	0.036	0.1–24.2	This work
CB/PLA 3DE (Grid) – EC	Amperometry	0.015	0.03–24.2	

Key: CFE, electrospun carbon nanofiber; CPE, carbon paste electrode; GMS, graphitic mesoporous carbon; GCE, glassy carbon electrode; NCNT, N‐doped carbon nanotube; CNFs, carbon nano fiber; GSEC, graphene sheets embedded carbon; 3D‐OPPy, 3D over‐oxidized polypyrrole; ERGO, reduced graphene oxide; CCE, ceramic carbon electrode; TCpSi, thermally carbonized porous silicon; SA‐900, 3D hierarchically porous carbon; CB‐PLA, carbon‐black polylactic acid; HC, hemicellulose‐derived carbon; LC, lignin‐derived carbon; KB, ketjen black; CB, carbon balck; PLA, polylactic acid; 3DE, 3D printed electrode; EC, electrochemically activated.

### 2.8 Reproducibility, Stability, Selectivity, and Real Sample Analysis

The interelectrode reproducibility of grid and compact electrodes was tested over the current response in the presence of 1 µM hydroquinone with five different electrodes. The relative standard deviation (RSD) for the grid and compact electrode was calculated to be 7.46% and 5.77%, respectively. This shows a high level of consistency in the current response over hydroquinone presence while also highlights a good reproducibility in what concerns the printing process and the activation method. The function of the activated sensor over a period of 10 days was assessed and the current response upon the addition of 1 µM hydroquinone is presented in Figure S10 (Supporting Information). The signal magnitude exhibited only minor variations over the 10‐days period, indicating excellent short‐term stability of the sensors.

Furthermore, the selectivity of the CB/PLA 3D printed electrodes for the amperometric determination of hydroquinone was evaluated by measuring the amperometric response of the addition of hydroquinone, followed by consecutive additions of possible interfering species. The results in Figure S11 (Supporting Information) indicate that the additions of 100 µΜ of urea, thiourea, and rhodamine 6G, 80 µΜ of caffeine, 50 µΜ of p‐nitroaniline and 1,3,5 trinitrobenzene, and 30 µΜ of p‐benzoquinone lead to signal responses less than 10% of the response obtained from 1 µM of hydroquinone. This indicates an exceptional selectivity of the proposed analytical method over the amperometric determination of hydroquinone and allows its use in real samples.

The reliability of EC activated CB/PLA 3D printed electrodes and the accuracy of the method were investigated through recovery studies in untreated tap water samples, for both grid and compact electrodes. The amperometric determination of hydroquinone in non‐spiked and 25 µM spiked samples for grid (Figure 6C) and compact (Figure 6D) electrodes was performed using the standard addition method. The recovery values of 106.4% and 101.3% (**Table** **2**) confirm the suitability of EC activated CB/PLA 3D printed electrodes for hydroquinone determination in tap water samples.

**Table 2:** Recovery of hydroquinone in spiked tap water samples.

Sample	Electrode	Added [µM]	Found [µM]	Recovery [%]
Tap Water	Grid	0	N.D^a^ ^)^	– 106.4
		25	26.59	
	Compact	0	N.D	– 101.3
		25	25.34	

^a)^ Not Detected.

## 3 Conclusion

In this work, CB/PLA 3D printed electrodes were investigated over their suitability for implementation in energy storage and sensing systems upon a single‐step EC treatment, using eco‐friendly and biocompatible conditions. The findings shed light on the impact of the treatment on the electrodes' performance. At the same time, the comparison between the two different electrode layouts (compact and grid) offers valuable insights into the influence of cavities in 3D printed electrodes.

Experimental data demonstrate that EC treatment induces the exposure of CB over PLA, enhancing the conductivity of the electrode's interface and resulting in uniform electrochemical activity across its surface. This is able to unlock favorable electrochemical properties, making them function as platforms for aqueous‐based electrochemical applications. At the same time, by simply adjusting the duration of the EC treatment the electrochemical behavior of the electrodes can be controlled and manipulated.

Both the EC activation and the electrode layout influence the performance of the CB/PLA 3D printed electrodes in different ways and to varying degrees, depending on the application. The EC treatment is able to enhance the intercalation kinetics in Zn‐ion batteries and significantly improve the sensitivity of the electrode toward diverse analytes, without promoting electrocatalytic water splitting reaction that can compromise the stability of the potential window and hence the sensing and energy storage performance.

Furthermore, the two types of electrodes show quite similar performance in the case of Zn‐ion batteries, with the compact retaining a slight advantage, while grid electrodes offer favorable sensing performance for most of the tested analytes and show higher sensitivity in what concerns the amperometric determination of hydroquinone. In particular, grid electrodes display a dynamic linear range of 0.03–24.2 µM with an LOD of 14.8 nM, while the compact electrodes exhibit a dynamic linear range of 0.1–24.2 µM with an LOD of 36.4 nM. Nevertheless, both of the electrodes offer competitive analytical figures of merit upon EC activation, along with exceptional storage stability, good reproducibility and reliability, that enable their implementation in real sample analysis.

These findings highlight the diversity of electrochemical applications proving that there is no universal solution when it comes to fabrication and activation of 3D printed electrodes, indicating the necessity of exploration every single time.

All in all, EC‐activated CB/PLA 3D printed electrodes offer improved efficiency and reliability in an economically viable manner. Thus, meet the increasing demand for developing sustainable and high‐performance energy storage and sensing platforms. The combination of eco‐compatible nature of the activation method with the flexibility of the grid layout electrode holds promise for future implementation of EC‐activated CB/PLA 3D printed electrodes in self‐powered wearable diagnostic devices. This advancement simplifies fabrication procedures and eliminates the need for multiple materials and technologies, in such devices.

## 4 Experimental Section

### Materials

CB/PLA filament was obtained from Protopasta (USA). Potassium hexacyanoferrate (III) (P > 98%), zinc sulfate heptahydrate (P > 99%), potassium chloride (P > 99%), ascorbic acid (P > 99.5%), urea (P > 99.5%) and thiourea (P > 99%) were products of Penta (Czech Republic), disodium hydrogen phosphate (P > 99%), potassium hexacyanoferrate (II) trihydrate (P > 98%), and sodium hydroxide (P > 97%) were products of Lachner (Czech Republic). Hydroquinone (P > 99%), epinephrine (P > 99%) and caffeine (P > 99%) were purchased from Sigma–Aldrich (USA), 1,3,5‐trinitrobenzene (P > 98%), p‐nitroaniline (P > 99.5%) and rhodamine 6G (P > 99%) from Lachema (Czech Republic), serotonin hydrochloride (P > 98%) and p‐benzoquinone (P > 99%) from Fluorochem (UK). PBS was prepared by mixing disodium hydrogen phosphate (0.1 M) and potassium chloride (0.1 M).

### Electrode Fabrication

The 3D printed electrodes were designed using sharp 3D software, sliced with Prusa Slicer software and printed by a Prusa Mini under the following settings, bed temperature: 60 °C, nozzle temperature: 215 °C, printing speed: 50–80 mm s^−1^, printed layer thickness: 0.1 mm and infill: 100%. The electrodes were activated by applying +1.8 V (versus Ag/AgCl) for 900 s in 0.1 M PBS (pH 7) unless stated otherwise.

### Mechanical Behavior

Mechanical test of the compact and grid 3D printed electrodes fabricated by CB/PLA and plain PLA was conducted with a tensile testing machine (Dongguan Zonhow Test Equipment, China) connected to data acquisition software for real‐time recording of force and elongation. The rectangular part of the electrode was placed between the upper and lower grips of the tensile testing machine with a gauge length of 3 mm and the compression test of the samples carried out with a speed of 0.5 mm min^−1^.

### Characterizations

Raman spectra were obtained using inVia Raman spectroscope (Renishaw, UK) in backscattering geometry equipped with a charge‐coupled device detector using DPSS green laser (532 nm, 50 mW), with 5 mW applied power and 20x magnification objective. FTIR measurements were performed on an iS50R FTIR spectrometer (Thermo Scientific, USA). The measurements were performed with a diamond ATR crystal, DLaTGS detector and KBr beamsplitter in the range from 4000 to 400 cm^−1^ at a resolution of 4 cm^−1^.

The morphology of the samples was investigated by SEM (Tescan Lyra 3 dual beam microscope). The non‐activated electrode and the EC‐activated electrodes were rinsed with water and dried under ambient conditions. The connection part of the electrode was cut off so only the rectangular working surface area would be placed on a conductive carbon tape. The SEM measurements were carried out with a secondary electron (SE) detector using a 5 kV electron beam for low magnification images and 10 kV for higher magnification images. The elemental composition of the electrodes’ surface was investigated through XPS utilizing a Phoibos 100 (Specs, Germany) with a monochromatic Al source (Kα1 = 1486.7 eV). The samples were placed on a conductive copper tape and compensated for with a flood gun to yield C 1s position at 284.8 eV. Survey spectra were recorded (Epass = 50 eV, step = 1 eV), followed by high‐resolution core‐level spectra of C 1s and O 1s regions (Epass = 20 eV, step = 0.1 eV). The peak fitting procedure was carried out in CasaXPS software.

Electrochemical mapping was obtained by SECM performed on a (Sensolytics GmbH, Germany) by a Pt ultramicroelectrode of 10 µm diameter with an increment of 5 µm in a total area of 100 µm^2^ of the compact CB/PLA 3D printed electrode by approaching the surface of the sample and measuring the changes in Faraday current at steady–state during the redox transition of 10 mM hexacyanoferrate (II) in 0.1 M KCl. A Pt wire served as the counter electrode, while the ultramicroelectrode that served as the working electrode was kept at a constant potential of 0.5 V versus Ag/AgCl reference electrode.

### Electrochemical Measurements

Electrochemical measurements were conducted using a Corrtest 4‐channel potentiostat in a three‐electrode electrochemical cell at room temperature. As‐printed or activated 3D printed electrodes were used as working electrodes at all times. Pt foil served as a counter electrode in electrochemical characterization (through CV and EIS) and sensing (through CV and amperometry), while a carbon rod served as a counter electrode in LSV, in all cases Ag/AgCl, 3 M KCl was used as a reference electrode, and all potentials reported hereafter refer to this electrode unless stated otherwise.

EIS was performed in the frequency range from 100 kHz to 0.1 Hz, and the amplitude of the applied AC potential was 5 mV, superimposed on a DC potential of 0.2 V. CV was measured with a scan rate of 25 mV s^−1^ unless stated otherwise. LSV was measured in 0.1 M PBS with a scan rate of 10 mV s^−1^ and amperometry experiments were conducted in stirred (150 rpm) solutions at a potential of +0.3 V.

All the above‐mentioned electrochemical measurements were conducted in 0.1 M PBS at pH 7 under ambient conditions, except for the electroreduction of 1,3,5‐trinitrobenzene in which the electrolyte was deoxygenated through Ar purging for 15 min prior to the measurements.

Zn electrochemistry measurements were carried out by CV at a scan rate of 0.1 mV s^−1^, utilizing CB/PLA 3D printed electrodes as cathode, a Zn‐foil as anode and 2.0 M ZnSO_4_ in distilled water served as the electrolyte.

### Real Sample Analysis

For the recovery experiments, a 4.875 mL tap water sample without any pretreatment was fortified with 0.125 mL of a 10^−3^ M hydroquinone stock solution resulting in a 25 µM spiked sample. Then 250 µL of the 25 µM hydroquinone‐spiked tap water sample was added to a 10 mL electrochemical cell (40‐fold dilution) and followed by three consecutive additions of 1 µM hydroquinone. The concentration of the analyte was calculated employing the standard addition method. The same procedure was followed for the determination of the analyte in the non‐spiked sample of tap water. The recovery values were determined by comparing the measured concentration of hydroquinone with the expected concentration.

## Conflict of Interest

The authors declare no conflict of interest.

## Supporting information



Supporting Information

## Data Availability

The data that support the findings of this study are available from the corresponding author upon reasonable request.
