# Supplementary material for: Unraveling the Versatility of Carbon Black – Polylactic Acid (CB/PLA) 3D‐Printed Electrodes via Sustainable Electrochemical Activation
Source: Small Methods. 2025 Mar 13;9(9):2402214. doi: 10.1002/smtd.202402214 (PMC12464624; doi:10.1002/smtd.202402214)
Supplement: Supplementary file 1 — Supporting Information [file SMTD-9-2402214-s001.docx]

Supporting Information

Unraveling the Versatility of Carbon Black - Polylactic Acid (CB/PLA) 3D-Printed Electrodes via Sustainable Electrochemical Activation

Anastasios V. Papavasileiou*, Lukáš Děkanovský, Levna Chacko, Bing Wu, Jan Luxa, Jakub Regner, Jan Paštika, Dana Koňáková, Zdeněk Sofer*


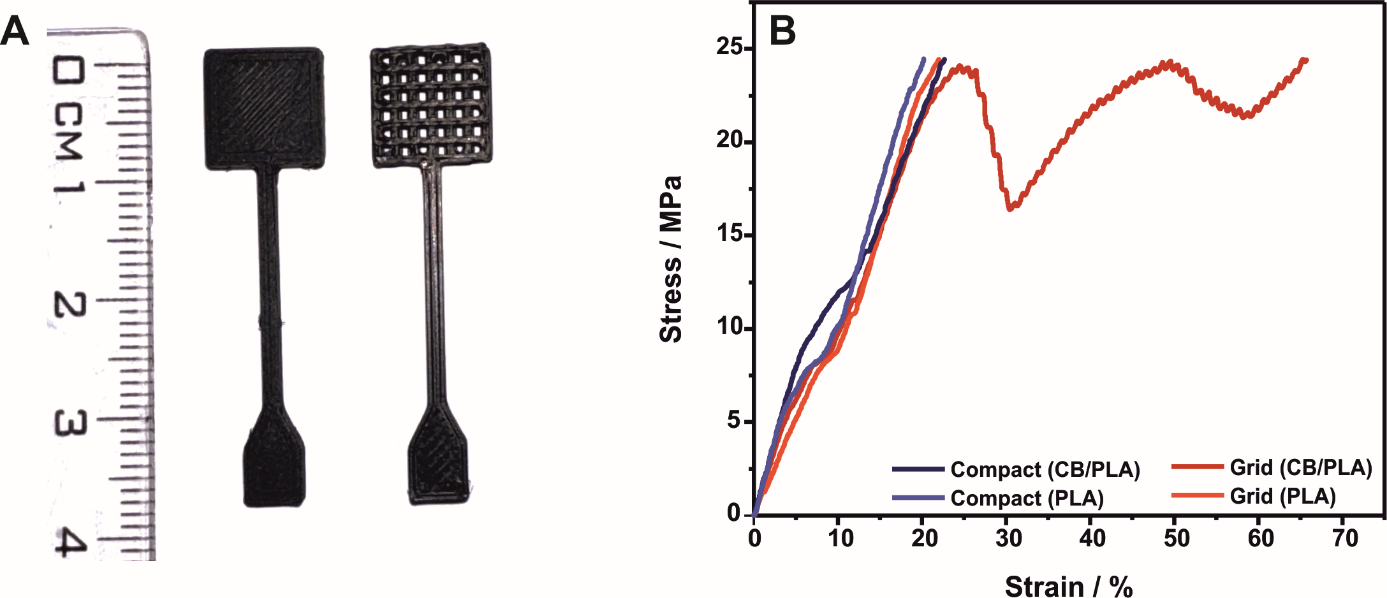


**Figure S1.** (A) Photo of the compact and grid CB/PLA 3D printed electrodes and (B) strain vs stress curves during the compression test of compact and grid 3D printed electrodes fabricated by CB/PLA and plain PLA.


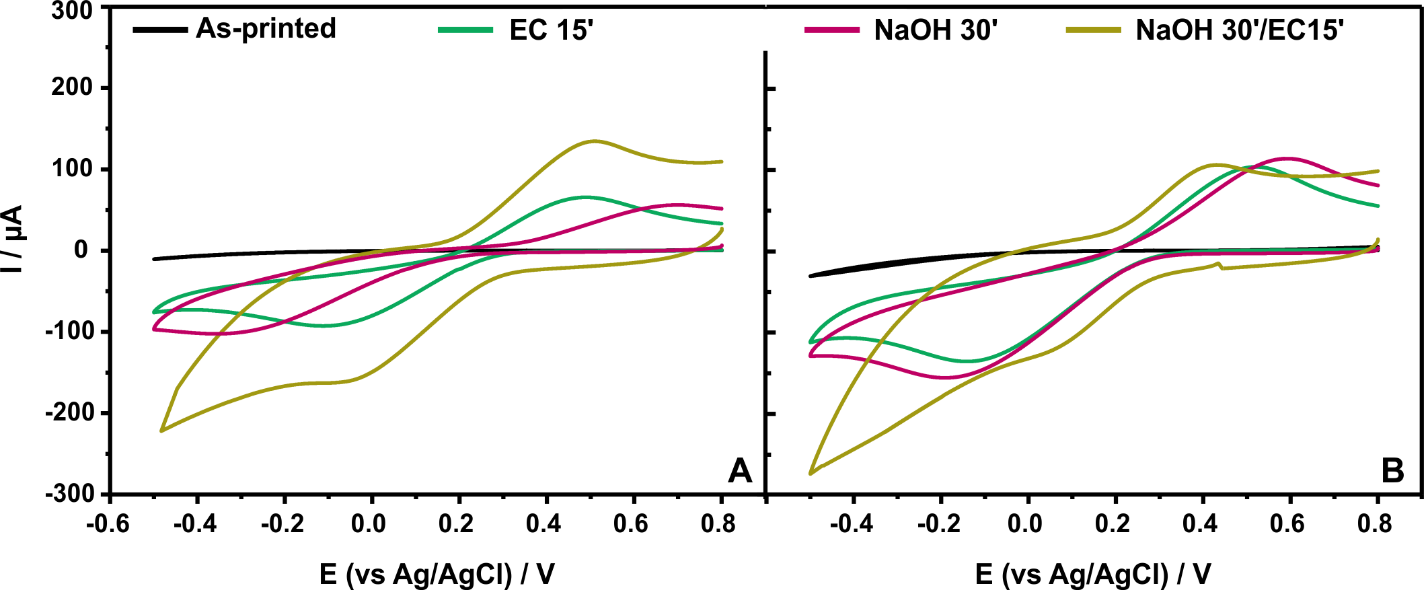


**Figure S2.** CVs of CB/PLA 3D compact (A) and grid (B) printed electrodes in 0.1 M PBS (pH 7) containing 0.5 mM hexacyanoferrate (III) at a scan rate of 50 mV s^−1^, before and after activation by different methods (chemical, electrochemical and combination of chemical/electrochemical).

| Printing Material | Activation Method | Activation Conditions | Advantages of the method | Disadvantages of the methods | Proposed Application | Performance | Ref. |
| --- | --- | --- | --- | --- | --- | --- | --- |
| Graphene/PLA | Chemical / Electrochemical | i) 1.0 M NaOH/30΄  ii) 0.1 M PBS (pH 6)/+1.8 V for 15΄ | -Ease-of-operation | -Scalability  -Hazardous wastes  -Multistep process | Dopamine Sensing | LDR 3-140 μM  LOD 1.44 μM | [S1] |
| Graphene/PLA | Mechanical | 30 s polishing with 1200 grit abrasive paper | -Reusability  -Ease-of-operation  -Rapid procedure  -Single step-process  -Sustainability | -Reproducibility  -Surface inhomogeneity  -Scalability | 2,4,6 trinitrotoluene sensing | LDR 1-870 μΜ  LOD 0.40 μM | [S2] |
| Graphene/PLA | Chemical / Electrochemical | i) DMF for 10΄  ii) 2.5 V for 150 s | -Rapid processing  -Ease-of-operation | -Scalability  -Toxic chemicals and hazardous wastes  -Mechanical integrity  -Chemical compatibility  -Multistep process | Hydrogen Evolution Reaction | Improves HER by overpotential (-1.5 mA cm^2^) of ca, 600 mV | [S3] |
| Graphene/PLA | Chemical | DMF for 10΄ | -Rapid processing  -Ease-of-operation  -Single step-process | -Toxic chemicals and hazardous wastes  -Mechanical integrity  -Chemical compatibility | Picric acid and ascorbic acid sensing | PA LDR 21.8-1527.7 μM  AA LDR 10-500 μM | [S4] |
| Graphene/PLA and CB/PLA | Chemical | THF for 5’ | -Rapid processing  -Ease-of-operation  -Single step-process | -Toxic chemicals and hazardous wastes  -Mechanical integrity  -Chemical compatibility | FcMeOH sensing | LDR 1-200 μM  LOD 1.32 μM | [S5] |
| CB/PLA | Electrochemical | 0.5 M NaOH / +1.4 V for 200 s followed by -1.0 V for 200 s | -Rapid processing  -Ease-of-operation  -Mechanical integrity  -Single step-process  -Reproducibility | -Scalability  -Hazardous wastes | Hydroxychloroquine sensing | LDR 0.4-7.5 μM  LOD 0.04 μΜ | [S6] |
| CB/PLA | Mechanical / Chemical-Electrochemical | 600 and 1200 grit sandpaper,  0.5 M NaOH / +1.4 V for 200 s followed by -1.0 V for 200 s | -Rapid processing | -Reproducibility  -Surface inhomogeneity  -Scalability  -Multistep process | Dopamine Sensing | LDR 1-250 μM  0.1 μM | [S7] |
| CB/PLA | Laser ablation | 1064 nm with a 6 s pulse duration (pulsed LaserBlast 500 Nd:YAG laser) | -Rapid processing  -Single step-process  -Mechanical integrity  -Biocompatibility | -Surface inhomogeneity  -Scalability  -Cost of instrumentation | Caffeine sensing | LDR 0.01 – 1 mM  LOD 0.4 μM | [S8] |
| CB/PLA | Spark-discharge | 1.2 kV in the presence of an external capacitor (5.3 nF), / 30’’ | -Rapid processing  -Sustainability  -Single step-process | -Surface inhomogeneity  -Scalability | Dopamine and Serotonin sensing | DP  LDR 1-10 μM  LOD 0.6 μM  5-HT  LDR 1-10 μM  LOD: 0.9 μM | [S9] |
| CB/PLA | Electrochemical | 0.1 M PBS pH 7/ +1.8 V for 15΄ | -Sustainability  -Surface Homogeneity  -Ease-of-operation  -Single step-process  -Mechanical integrity  -Biocompatibility | -Scalability | Hydroquinone sensing | Grid  LDR 0.03 -24.2  LOD 0.015  Compact  LDR 0.1-24.2  LOD 0.036 | This work |

**Table S1.** An overview of the activation strategies of 3D printed electrodes based on the electrode material to which they were applied.


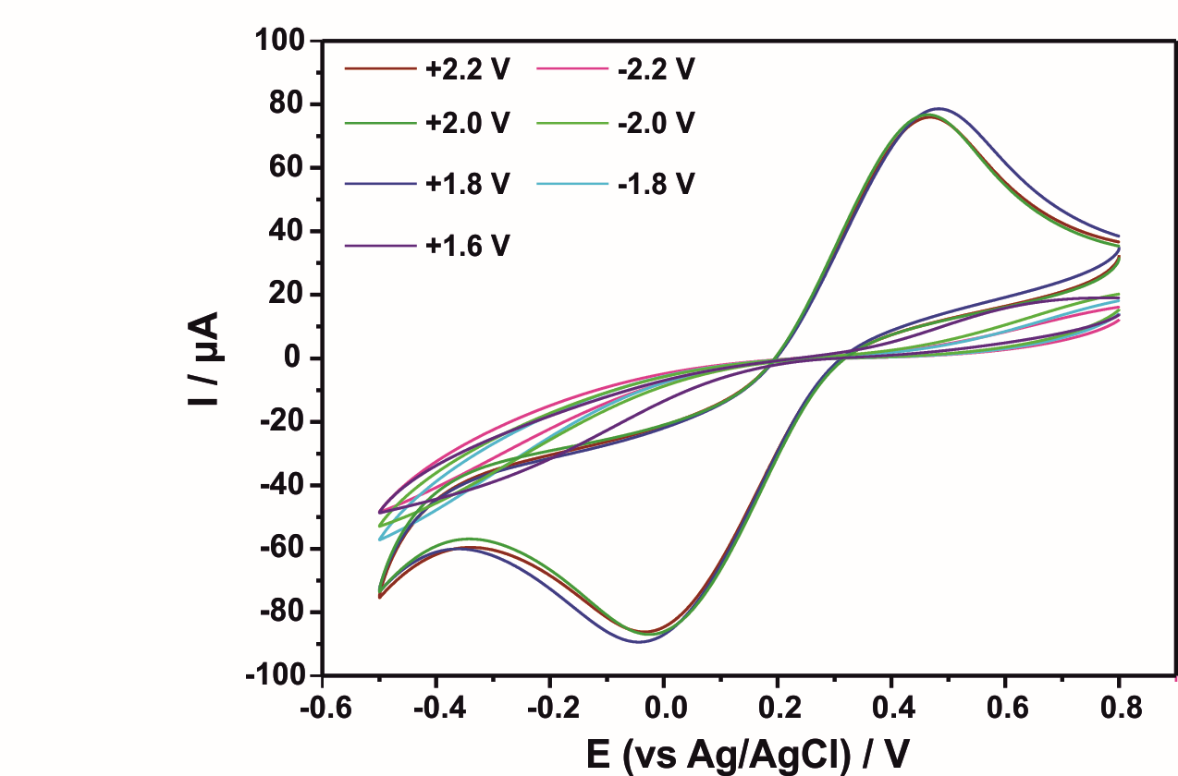


**Figure S3.** Cyclic Voltammograms of CB/PLA 3D printed electrodes in 0.1 M PBS (pH 7) containing 0.5 mM hexacyanoferrate (III) at a scan rate of 25 mV s^−1^, after EC activation for 900 s at various anodic and cathodic potentials.


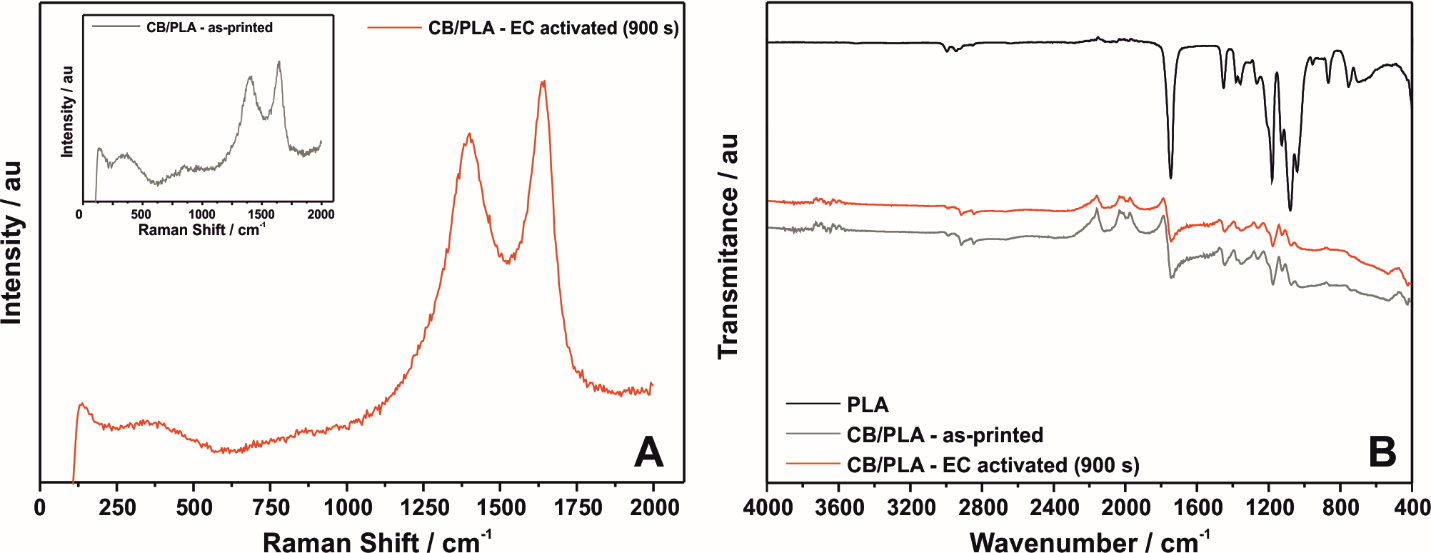


**Figure S4**. Raman spectra (A) and FTIR transmittance spectra (B) of CB/PLA 3D printed electrodes before and after EC activation.


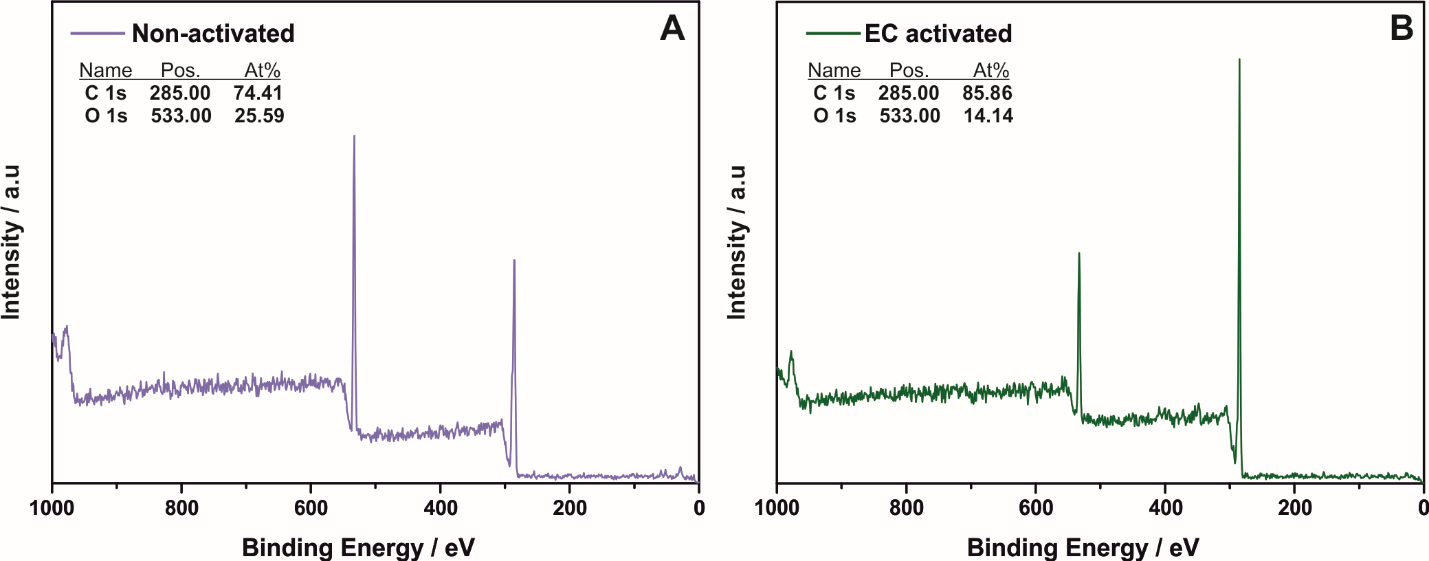


**Figure S5**. XPS survey spectra for non-activated (A) and EC activated (B) 3D printed electrodes.

**
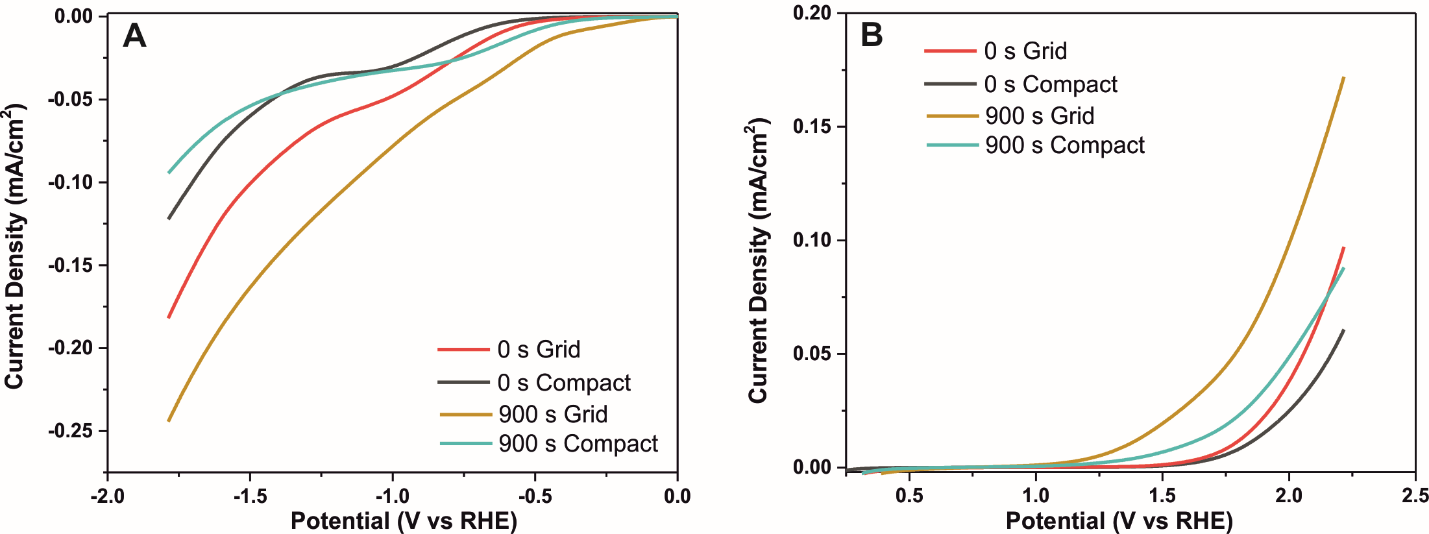
**

**Figure S6.** LSV plots of HER (A) and OER (B) of CB/PLA 3D printed electrodes for different electrode layouts before and after activation.

Zn Electrochemistry

Zn-ion batteries have been drawing a large interest thanks to their compatibility with aqueous electrolytes that introduces a new level of safety and eco-friendliness. Additionally, Zn-ion batteries are promising candidates thanks to the relatively low redox potential, low cost, high safety, high theoretic capacity, and excellent performance of Zn as anode material.^[S10, S11]^ The divalent nature of Zn is promising for higher specific capacity in comparison with monovalent ions, and its natural abundance renders it a plausible alternative to Li-ion batteries.^[S12]^ To this end, the effectiveness of a 3D printed CB/PLA electrode as a cathode for a Zn-ion battery was assessed. This study was carried out through CV in a 2 M ZnSO_4_ aqueous electrolyte at a scan rate of 0.1 mV s^−1^ with a Zn foil serving as anode (**Figure S7 A**). The cycling behavior of compact and grid 3D printed electrodes is displayed in **Figure S7 C-F**, where the intercalation/deintercalation of the Zn ion is apparent. The good electrical conductivity of CB in combination with the ability of PLA to interact with Zn-ions through carbonyl/carboxyl groups^[S13]^ (as illustrated in **Figure S7 B)** result in CB/PLA-based electrodes uniquely function as active material without the necessity of additional active substances; exhibiting a high oxidation-reduction potential around 1.5 V vs. Zn/Zn^2+^. This distinguishes them as promising candidates for high energy-density cathode materials and ensuring superior energy storage capabilities. Upon EC activation, the oxidation peak shifts to lower potential values whilst the reduction peak position remains unaffected, leading to a lower oxidation-reduction distance, hence higher intercalation kinetics.^[S14]^


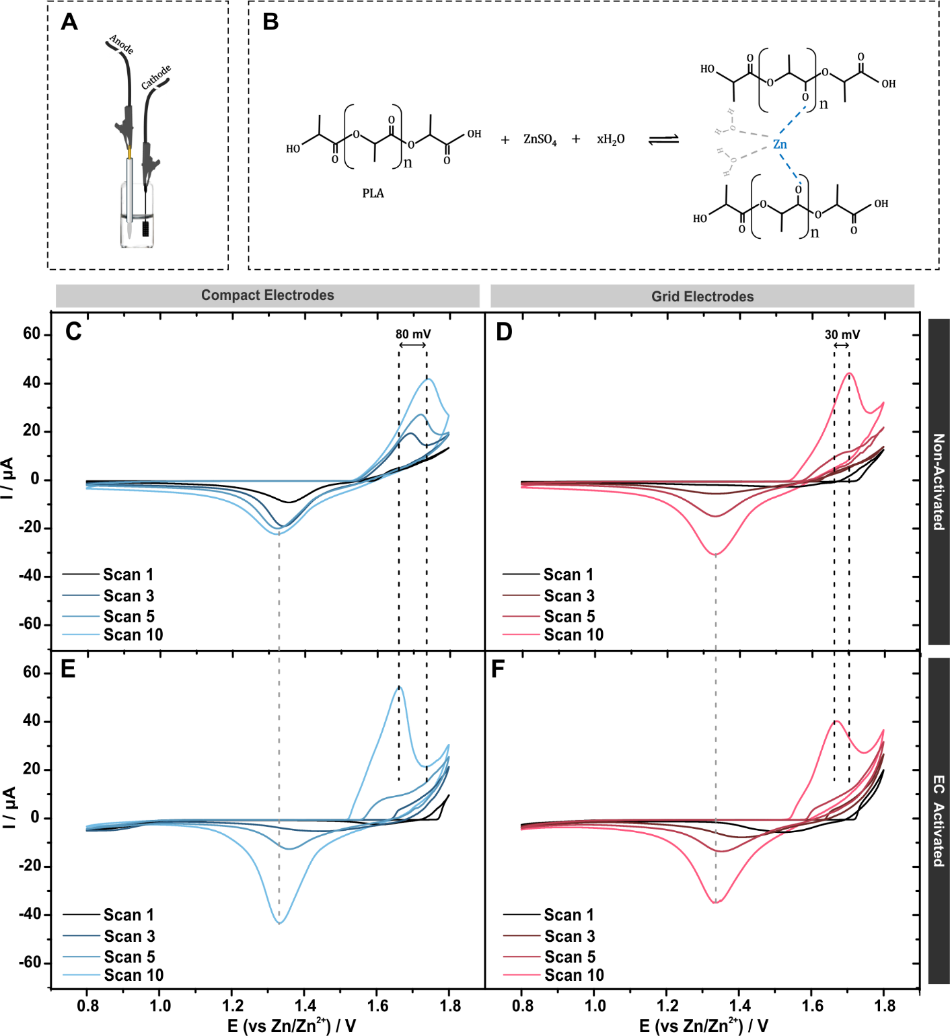


**Figure S7.** (A) Graphical illustration of the assessed Zn-ion battery assembly and (B) possible mechanism that undergoes the charge-discharge reaction. CVs in which CB/PLA 3D printed compact (C, E) and grid (D, F) electrodes before (C-D) and after (E-F) EC activation served as cathode and Zn foil as anode in a ZnSO_4_ electrolyte at a scan rate of 0.1 mV s^−1^.

As demonstrated in **Figure S7 C-F**, the cathodic peak potential of the non-activated electrode is constant in the different scans, while in the activated electrode, it appears in a higher potential value and gradually shifts until it becomes similar to the non-activated electrode. Compact electrodes are more effective thanks to the continuous network of PLA that interacts with Zn ions. At the same time, CB/PLA 3D printed electrodes display a remarkable cycling stability over 100 scans (**Figure S8**), maintaining 90% capacity retention, which is indicative of excellent long-term performance.

However, prior the full implementation of CB/PLA 3d printed electrodes in energy storage applications, there are several challenges to be addressed. The increased resistivity of the electrodes, as occurs from the EIS data attributed to the limited amount of percolation paths in the CB/PLA composite, is expected to lead to a high iR drop and, consequently, to energy loss.

In order to ameliorate the expected iR drop issues and enhance the performance of CB/PLA 3D printed electrodes in energy storage applications, several strategies can be employed. One simple way is by reducing the thickness and/or length of the electrode. This will reduce the contact resistance and the conductive pathway, easing the flow of the current.^[S15]^ Another way is to enrich the filament with a higher amount of carbon black or add another conductive material to increase the amount of percolation paths.^[S16, S17, S18]^


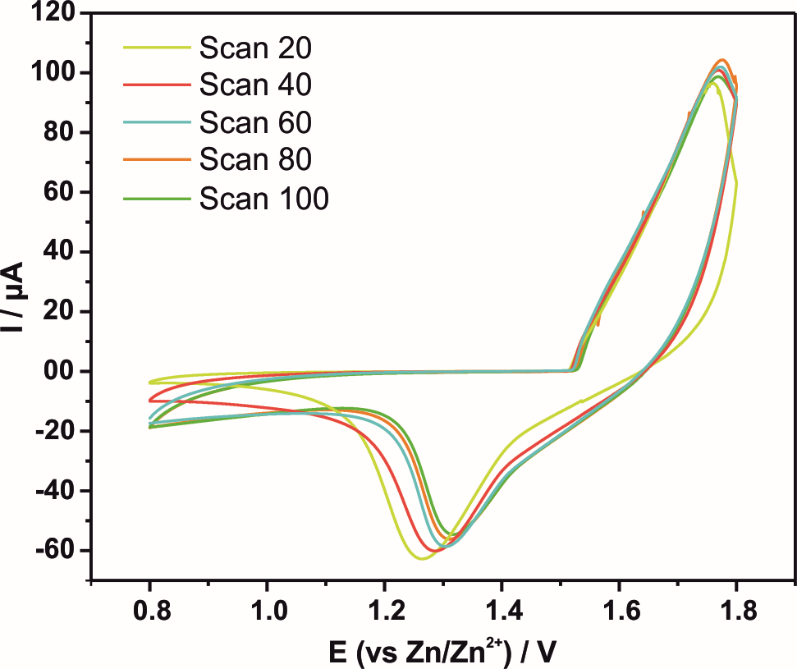


**Figure S8.** CVs of EC activated CB/PLA 3D printed electrodes of 100 scans of charge-discharge of Zn ions measured with a Zn foil as an anode in a ZnSO_4_ as electrolyte at a scan rate of 0.1 mV s^−1^.

**
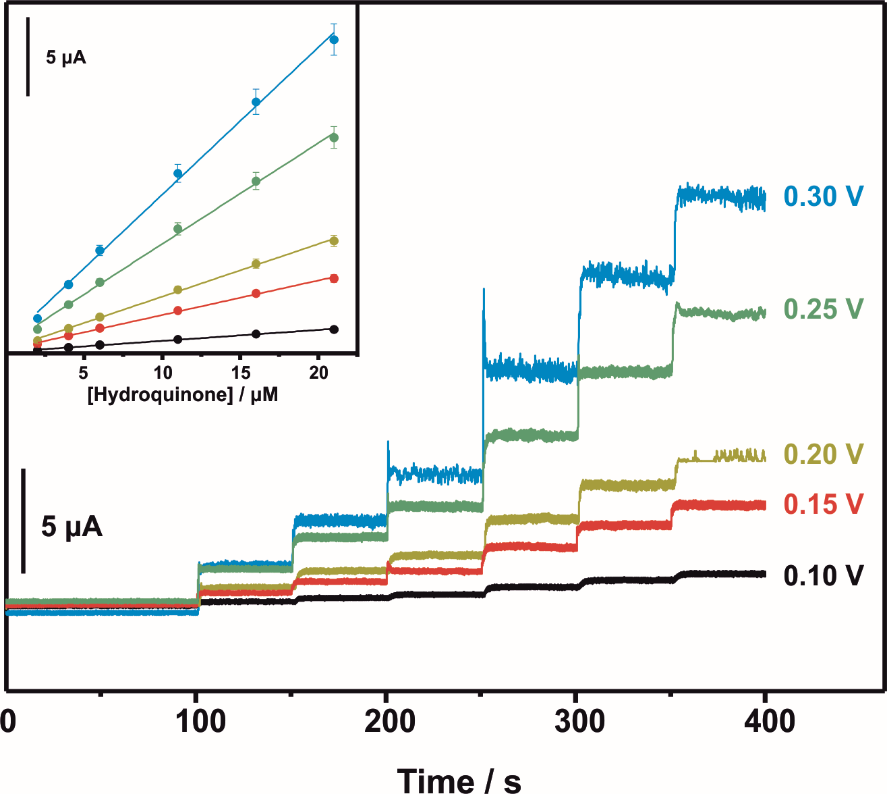
**

**Figure S9.** Amperometric curves recorded on EC activated CB/PLA grid 3D printed electrodes at various polarization potentials from +0.1 to +0.3 V in a stirred (150 rpm) 0.1 M PBS (pH 7) electrolyte tracking the response over six consecutive additions of hydroquinone (i.e. 3 x 2 μΜ, 3 x 5 μM). The inset graph displays the respective calibration plots.

**
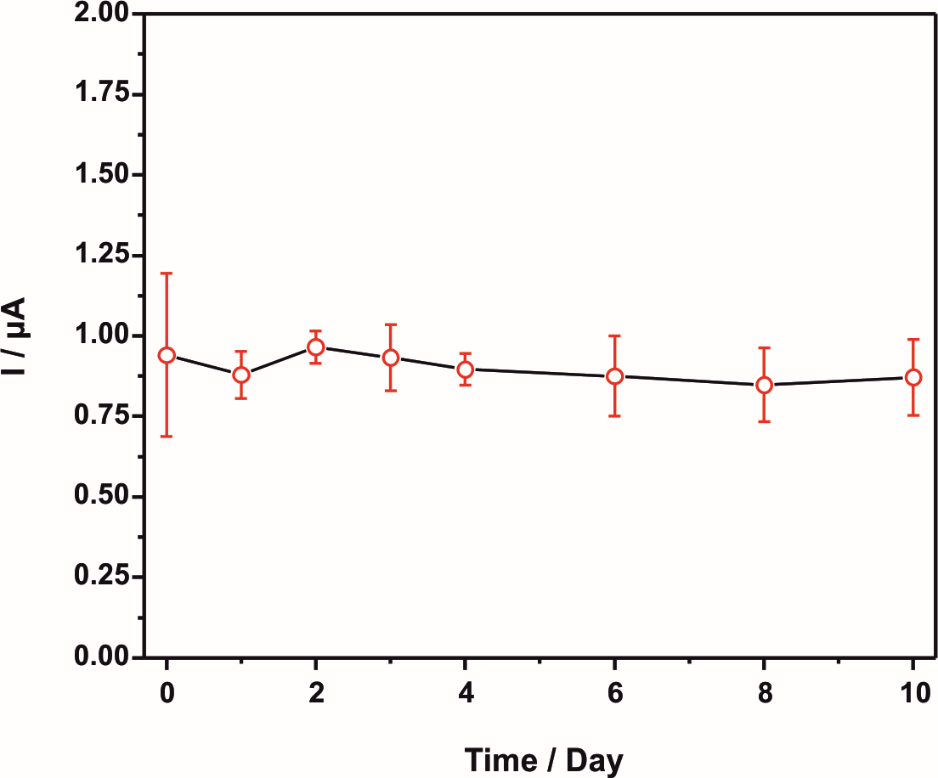
**

**Figure S10.** Response of the EC-activated CB/PLA grid electrodes upon the addition of 1 μM hydroquinone in a 0.1 M PBS (pH 7) over a period of 10 days.

**
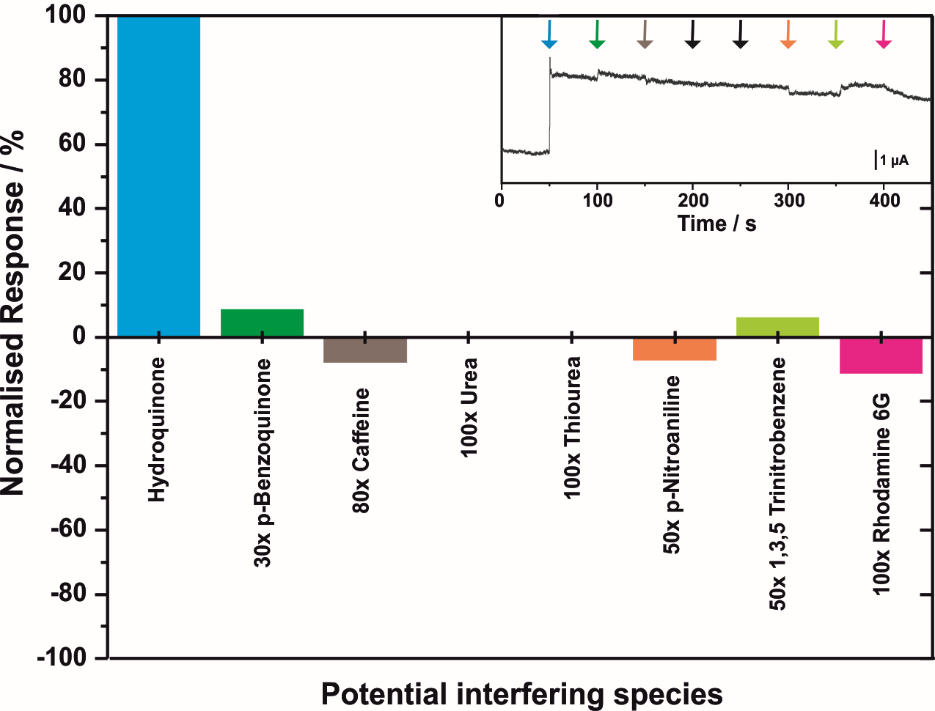
**

**Figure S11.** Selectivity test of EC activated CB/PLA 3D printed electrodes over the presence of excess concentration of potential interfering species. The bars demonstrate the percentage of the signal response of the potential interferents to 1 μM of hydroquinone. The inset graph illustrates the amperometric curve of the selectivity test where the addition of 1 μM hydroquinone is followed by 30 μM p-benzoquinone, 80 μM caffeine, 100 μM urea, 100 μM thiourea, 50 μM p-nitroaniline, 50 μM 1,3,5-trinitrobenzene and 100 μM rhodamine 6G.

**References**

(S1) Kalinke, C.; Vacilotto Neumsteir, N.; de Oliveira Aparecido, G.; Vasconcelos de Barros Ferraz, T.; Layene dos Santos, P.; Campos Janegitz, B.; Alves Bonacin, J. Comparison of Activation Processes for 3D Printed PLA-Graphene Electrodes: Electrochemical Properties and Application for Sensing of Dopamine. *Analyst* **2020**, *145*, 1207.

(S2) Cardoso, R. M.; Castro, S. V. F.; Silva, M. N. T.; Lima, A. P.; Santana, M. H. P.; Nossol, E.; Silva, R. A. B.; Richter, E. M.; Paixão, T. R. L. C.; Muñoz, R. A. A. 3D-Printed Flexible Device Combining Sampling and Detection of Explosives. *Sens Actuators B Chem* **2019**, *292*, 308–313.

(S3) Browne, M. P.; Novotný, F.; Sofer, Z.; Pumera, M. 3D Printed Graphene Electrodes’ Electrochemical Activation. *ACS Appl Mater Interfaces* **2018**, *10* (46), 40294–40301.

(S4) Manzanares Palenzuela, C. L.; Novotný, F.; Krupička, P.; Sofer, Z.; Pumera, M. 3D-Printed Graphene/Polylactic Acid Electrodes Promise High Sensitivity in Electroanalysis. *Anal Chem* **2018**, *90* (9), 5753–5757.

(S5) Kwaczyński, K.; Szymaniec, O.; Bobrowska, D. M.; Poltorak, L. Solvent-Activated 3D-Printed Electrodes and Their Electroanalytical Potential. *Sci Rep* **2023**, *13*, 22797.

(S6) Carvalho, M. S.; Rocha, R. G.; De Faria, L. V; Richter, E. M.; Dantas, M. F.; Da Silva, I. S.; Muñoz, R. A. A. Additively Manufactured Electrodes for the Electrochemical Detection of Hydroxychloroquine. *Talanta* **2022**, *250*, 123727.

(S7) Richter, E. M.; Rocha, D. P.; Cardoso, R. M.; Keefe, E. M.; Foster, C. W.; Munoz, R. A. A.; Banks, C. E. Complete Additively Manufactured (3D-Printed) Electrochemical Sensing Platform. *Anal Chem* **2019**, *91* (20), 12844–12851.

(S8) Glowacki, M. J.; Cieslik, M.; Sawczak, M.; Koterwa, A.; Kaczmarzyk, I.; Jendrzejewski, R.; Szynkiewicz, L.; Ossowski, T.; Bogdanowicz, R.; Niedzialkowski, P.; Ryl, J. Helium-Assisted, Solvent-Free Electro-Activation of 3D Printed Conductive Carbon-Polylactide Electrodes by Pulsed Laser Ablation. *Appl Surf Sci* **2021**, *556*, 149788.

(S9) Hernández-Rodríguez, J. F.; Trachioti, M. G.; Hrbac, J.; Rojas, D.; Escarpa, A.; Prodromidis, M. I. Spark-Discharge-Activated 3D-Printed Electrochemical Sensors. *Anal. Chem.* **2024**, *96* (25), 10127–10133.

(S10) Kang, J.; Zhao, Z.; Li, H.; Meng, Y.; Hu, B.; Lu, H. An Overview of Aqueous Zinc-Ion Batteries Based on Conversion-Type Cathodes. *Energy Mater.* **2022**, *2*, 200009.

(S11) Yuan, L.; Hao, J.; Kao, C. C.; Wu, C.; Liu, H. K.; Dou, S. X.; Qiao, S. Z. Regulation Methods for the Zn/Electrolyte Interphase and the Effectiveness Evaluation in Aqueous Zn-Ion Batteries. *Energy Environ. Sci.* **2021**, *14*, 5669–5689.

(S12) Fang, G.; Zhou, J.; Pan, A.; Liang, S. Recent Advances in Aqueous Zinc-Ion Batteries. *ACS Energy Lett.* **2018**, *3*, 2480–2501.

(S13) Yang, B.; Ma, Y.; Bin, D.; Lu, H.; Xia, Y. Ultralong-Life Cathode for Aqueous Zinc-Organic Batteries via Pouring 9,10-Phenanthraquinone into Active Carbon. *ACS Appl. Mater. Interfaces* **2021**, *13*, 58818–58826.

(S14) Stephen, A.; Bhoyate, S.; Cao, P.; Advincula, R.; Dahotre, N.; Jiang, Y.; Choi, W. 3D-Printed Flexible Anode for High-Performance Zinc-Ion Battery. *MRS Commun.* **2022**, *12*, 894–901.

(S15) Veloso, W. B.; Paixão, T. R. L. C.; Meloni, G. N. 3D-Printed Electrodes Design and Voltammetric Response. *Electrochim. Acta* **2023**, *449*, 142166.

(S16) Cieślik, M.; Susik, A.; Banasiak, M.; Bogdanowicz, R.; Formela, K.; Ryl, J. Tailoring Diamondised Nanocarbon-Loaded Poly(Lactic Acid) Composites for Highly Electroactive Surfaces: Extrusion and Characterisation of Filaments for Improved 3D-Printed Surfaces. *Microchim. Acta* **2023**, *190, 370*.

(S17) Caldas, N. M.; de Faria, L. V.; Batista, A. G.; Alves, A. O.; de Souza, C. C.; Borges, P. H. S.; Nossol, E.; Matos, R. C.; Rocha, D. P.; Semaan, F. S.; Dornellas, R. M. Lab-Created Conductive Filament Based on Nickel and Graphite Particles: An Attractive Material for the Additive Manufacture of Enhanced Electrochemical Sensors for Non-Enzymatic and Selective Glucose Sensing. *Talanta* **2025**, 287 *127686*.

(S18) Stefano, J. S.; Guterres e Silva, L. R.; Rocha, R. G.; Brazaca, L. C.; Richter, E. M.; Abarza Muñoz, R. A.; Janegitz, B. C. New Conductive Filament Ready-to-Use for 3D-Printing Electrochemical (Bio)Sensors: Towards the Detection of SARS-CoV-2. *Anal. Chim. Acta* **2022**, *1191, 339372*.
